# Neuromuscular embodiment of feedback control elements in *Drosophila* flight

**DOI:** 10.1126/sciadv.abo7461

**Published:** 2022-12-14

**Authors:** Samuel C. Whitehead, Sofia Leone, Theodore Lindsay, Matthew R. Meiselman, Noah J. Cowan, Michael H. Dickinson, Nilay Yapici, David L. Stern, Troy Shirangi, Itai Cohen

**Affiliations:** ^1^ Department of Physics, Cornell University, Ithaca, NY 14850, USA.; ^2^ Department of Biology, Villanova University, Villanova, PA 19805, USA.; ^3^ Division of Biology and Bioengineering, California Institute of Technology, Pasadena, CA 91125, USA.; ^4^ Department of Neurobiology and Behavior, Cornell University, Ithaca, NY 14850, USA.; ^5^ Department of Mechanical Engineering, Laboratory for Computational Sensing and Robotics, Johns Hopkins University, Baltimore, MD 21218, USA.; ^6^ HHMI Janelia Research Campus, Ashburn, VA 19700, USA.

## Abstract

While insects such as *Drosophila* are flying, aerodynamic instabilities require that they make millisecond time scale adjustments to their wing motion to stay aloft and on course. These stabilization reflexes can be modeled as a proportional-integral (PI) controller; however, it is unclear how such control might be instantiated in insects at the level of muscles and neurons. Here, we show that the b1 and b2 motor units—prominent components of the fly’s steering muscle system—modulate specific elements of the PI controller: the angular displacement (integral) and angular velocity (proportional), respectively. Moreover, these effects are observed only during the stabilization of pitch. Our results provide evidence for an organizational principle in which each muscle contributes to a specific functional role in flight control, a finding that highlights the power of using top-down behavioral modeling to guide bottom-up cellular manipulation studies.

## Teaser

In free-flying *Drosophila*, the wing basalar muscles b1 &amp; b2 actuate distinct control outputs of a rapid stabilization circuit.

## INTRODUCTION

To maintain stability, locomoting animals continuously update their motor actions based on sensory information (*1*, *2*). These motor corrections are particularly important during extreme forms of locomotion such as insect flight, where aerodynamic instabilities emerge rapidly when left uncorrected (*3*–*5*). To contend with these instabilities, insects such as *Drosophila* sense changes in their body orientation and respond with subtle modulations in wing motion on millisecond time scales (*6*–*9*). This feedback control underlying *Drosophila* flight can be modeled by a set of proportional-integral (PI) controllers that describe the stabilization of all three rotational degrees of freedom: yaw (*10*), pitch (*11*, *12*), and roll (*13*). These PI controller models linearly combine the fly’s body angular velocity (P) and its angular displacement (I) to quantitatively predict changes in wing motion that counteract perturbations. These control theoretic models offer a powerful framework for describing sensorimotor feedback rules and have been successfully applied to describe many behaviors across animals (*2*, *14*–*25*). Here, we combine this powerful top-down framework for describing behavior with bottom-up genetic tools for cell-specific manipulation (*26*, *27*) to elucidate the neuromuscular implementation of these flight stabilization reflexes in freely moving flies.

**Fig. 1.**
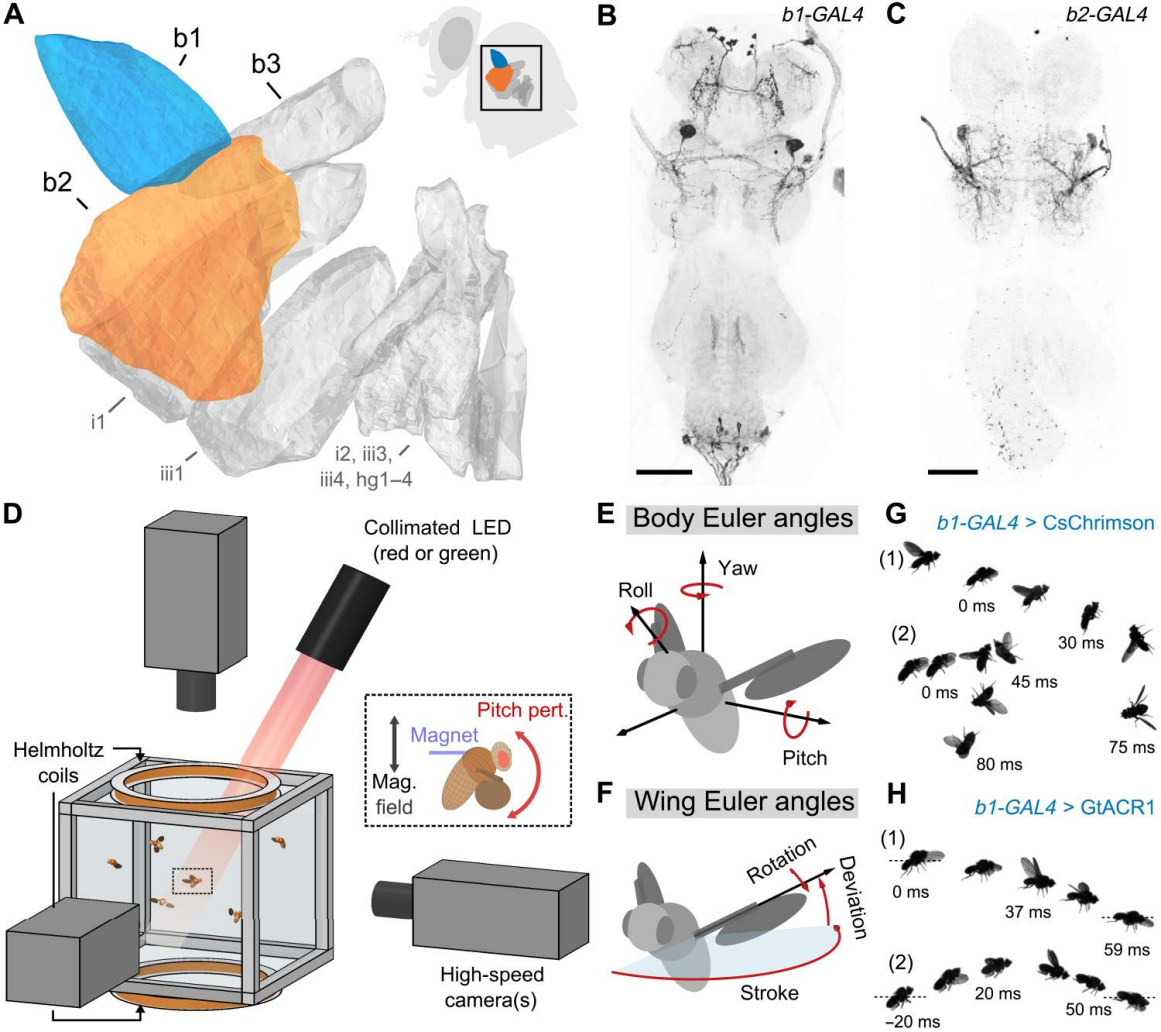
Combining genetic tools and free flight apparatus to quantify the effects of b1 and b2 manipulations. (**A**) Direct steering muscles of the *Drosophila* wing motor, with the first and second basalars (b1 and b2) highlighted in blue and orange, respectively. Data are from (*30*). The inset shows a fly silhouette, with a black box indicating the approximate position of the steering muscles. (**B** and **C**) Maximum intensity projections of the fly ventral nerve cord (VNC) expressing CsChrimson-mVenus (black) driven by *b1-GAL4* (B) and *b2-GAL4* (C). The light gray color shows DNCad (neuropil). Scale bars, 50 μm. (**D**) Schematic of the experimental apparatus used to deliver optogenetic and/or mechanical perturbations to freely flying *Drosophila* while filming their maneuvers at 8000 frames/s. The inset illustrates magnetic field from Helmholtz coils interacting with a magnetic pin glued to a fly to produce a perturbing pitch torque—data from these magnetic perturbation events are used to directly investigate the flight stabilization reflex. The optical trigger (not shown) allows automated capture of hundreds of movies per trial. (**E** and **F**) Definitions of the body (E) and wing (F) Euler angles used to describe flight kinematics. (**G** and **H**) Photomontages of example flies undergoing optogenetic excitation (G) (movies S1 and S2) and silencing (H) (movies S3 and S4) of the b1 motoneuron using CsChrimson and GtACR1, respectively. Each panel shows photomontages of two flies—labeled “(1)” and “(2)”—with time stamps indicating the timing relative to the onset of the 50-ms light-emitting diode (LED) stimulus (*t* = 0 ms). See table S2 for full fly genotypes.

The impressive aerial agility of flies such as *Drosophila* is made possible by the cleverly specialized musculature driving wing motion: Large, asynchronous muscles that fill the thorax provide the power for high-frequency wing strokes, while a set of 12 small, synchronous muscles (Fig. 1A)—each of which receives input from a sole excitatory motor neuron (*28*)—actuate subtle changes to wing kinematics on fast time scales, thereby implementing fast flight control (*29*, *30*). Two of these 12 steering muscles that are thought to play a prominent role in flight control are the first and second basalar muscles, b1 and b2 (Fig. 1A) (*30*–*34*). These muscles regulate wing motion via their agonistic actions on the basalar sclerite, a skeletal element at the base of the wing (*29*). Studies in *Drosophila* and *Calliphora* (blowflies) demonstrate that changes in either b1 or b2 muscle activity contribute to the modulation of wing stroke amplitude (*30*–*35*), a primary control parameter used to stabilize both roll (*13*) and pitch orientation (*12*). Despite their similar effects on wing kinematics, however, the b1 and b2 muscles differ markedly in their physiology: b1 is tonically active during flight and can encode changes in wing kinematics via phase shifts in firing, whereas b2 is phasically activated during maneuvers but is generally quiescent during straight flight bouts (*30*–*32*). Together, these studies suggest that b1 and b2 are both poised to play critical, but potentially distinct, roles in rapid flight control.

## RESULTS

To first resolve the effects of b1 and b2 manipulation on free flight kinematics, we measured changes in wing and body dynamics of flies experiencing brief bouts of midair optogenetic excitation or inhibition (Fig. 1 and Materials and Methods). In these experiments, we targeted the motoneurons of the b1 and b2 muscles using the split-GAL4 driver lines *MB258C-GAL4* (*b1-GAL4*; Fig. 1B; fig. S1, A and B; and table S1) and *b2-SG* (*b2-GAL4*; Fig. 1C; fig. S1, C and D; and table S1) (*36*) to drive the expression of CsChrimson (excitation) (*37*) or GtACR1 (inhibition) (*38*). Using the flight chamber shown in Fig. 1D, we captured and quantified flight kinematics (Fig. 1, E and F) before, during, and after the application of a 50-ms light pulse. Figure 1 (G and H) shows photomontages of responses to b1 motoneuron excitation (Fig. 1G) and inhibition (Fig. 1H) viewed from the side. As illustrated in Fig. 1G, excitation of the b1 motoneuron evoked extreme upward pitching maneuvers, with the fly rotating ≥90° during the 50-ms period of stimulation (movies S1 and S2). Under b1 motoneuron inhibition, flies pitched downward, dipping to angles below the horizontal plane (Fig. 1H and movies S3 and S4).

**Fig. 2.**
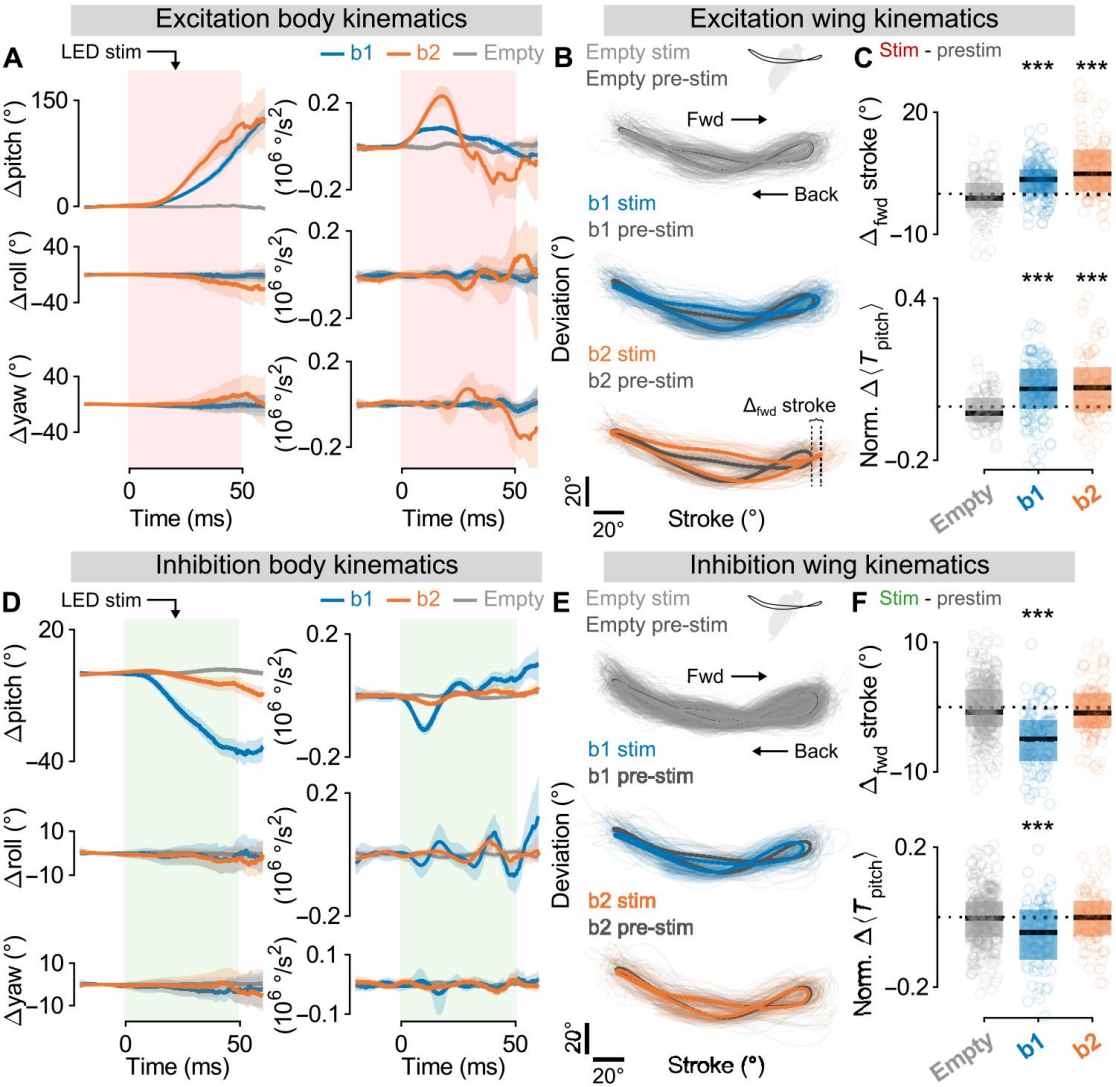
In-flight optogenetic activation and silencing of the b1 and b2 motoneurons drive changes to pitch orientation. (**A**) Body kinematics versus time in response to 50-ms optogenetic activation of *b1-GAL4* (blue; *n* = 140 movies), *b2-GAL4* (orange; *n* = 84 movies), and *SS01062-GAL4* (aka empty; gray; *n* = 108 movies) flies with CsChrimson. Rows correspond to rotational degrees of freedom: pitch (top), roll (middle), and yaw (bottom). Columns give angular displacement (left) and angular acceleration (right). Data shown represent means ± 95% confidence interval (CI). (**B**) Wing kinematic data averaged across the left and right wings for movies in (A). Plots show wing tip angular position in the wing strokes before (dark gray; pre-stim) and during (light gray, blue, and orange; stim) optogenetic activation. Thick traces represent population averages; thin lines represent single-fly wingbeats. Vertical and horizontal scale bars provide 20 references for deviation and stroke angles, respectively. (**C**) Change in forward stroke angle (“Δ_fwd_ stroke,” top) and normalized, wingbeat-averaged aerodynamic pitch torque (“norm. Δ〈*T*_pitch_〉,” bottom) for wingbeats before and during optogenetic activation of *SS01062-GAL4* (gray), *b1-GAL4* (blue), and *b2-GAL4* (orange) flies. Circles show raw data; box and horizontal line show interquartile range and median, respectively. As in (B), data are combined across the left and right wings. Statistical significance is determined via Wilcoxon signed-rank test (***P < 0.001). (**D** to **F**) Same as in (A) to (C), but with optogenetic silencing of *b1-GAL4* (blue; *n* = 89 movies), *b2-GAL4* (orange; *n* = 89 movies), and *SS01062-GAL4* (gray; *n* = 323 movies) flies with GtACR1. See table S2 for full fly genotypes.

To quantify these kinematic changes, we analyzed hundreds of these flight videos (Fig. 2). The optogenetic excitation of both *b1-GAL4* and *b2-GAL4* flies drove large, nose-up deviations in pitch—net pitch rotations of 90.5° ± 4.2° and 117.3° ± 13.9° for *b1-GAL4* and *b2-GAL4* flies, respectively (means ± SE)—with smaller deviations in roll and yaw (net rotations all <17° in magnitude) [Fig. 2A (blue and orange) and movies S5 and S6]. The large changes in pitch orientation were driven by bilateral modulations of wingbeat angles during stimulation (Fig. 2B). During stimulated wingbeats, we observed a statistically significant increase in the forward stroke angle (Fig. 2C, top) and a corresponding increase in the average aerodynamic pitch torque per wingbeat, estimated using a quasi-steady model [Fig. 2C (bottom) and Supplementary Text] (*39*). In contrast, genetic control experiments using the empty split-GAL4 line *SS01062-GAL4* (aka empty) (*40*) with the *UAS-CsChrimson* transgene showed no measurable changes in either body orientation (net average rotations in yaw, pitch, and roll are all <2° in magnitude) or wing motion upon optogenetic excitation [Fig. 2, A to C (gray), and movie S7]. Overall, our findings are qualitatively consistent with previous electrophysiological studies in flies, which showed that increased activity in either b1 or b2 drove marked increases in wing stroke parameters such as the downstroke deviation and forward stroke angles (fig. S2) (*31*–*34*).

Performing the same analyses with optogenetic silencing, we found that b1-silenced flies primarily underwent large, nose-down changes to pitch—comparable in magnitude to those observed during rapid escape responses (*41*)—while b2-silenced flies exhibited a smaller, but still noticeable, decrease in pitch attitude (net pitch rotations of −35.4° ± 2.0° and −5.9° ± 1.5° for *b1-GAL4* and *b2-GAL4* flies, respectively) [Fig. 2D (blue and orange) and movies S8 to S10]. Correspondingly, we observed the largest change in b1-silenced flies’ wing strokes (Fig. 2E, blue), with a significant decrease in both forward stroke angle and resulting wingbeat-averaged pitch torque as compared to both b2-silenced and the genetic control flies (Fig. 2F). The relatively small effect in b2-silenced flies is consistent with the fact that b2 is a phasic muscle (*30*) and, thus, would likely be quiescent during steady-state, unperturbed flight. Collectively, these results indicate that changes in bilateral b1 motoneuron activity are capable of bidirectionally modulating pitch torque, whereas bilateral manipulation of the b2 motoneuron activity results only in pitch up torque. This ability to affect pitch orientation confirms that both muscles could play an important role in the control of this degree of freedom.

**Fig. 3.**
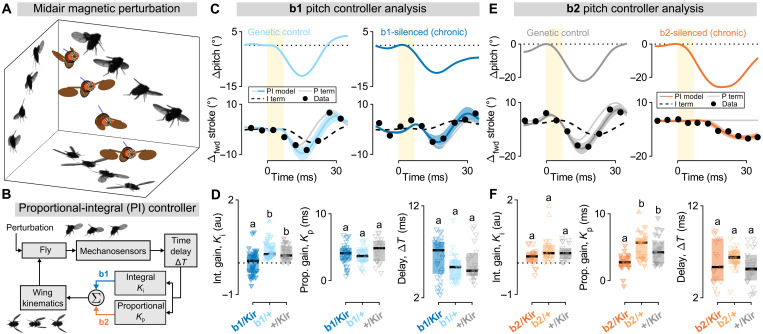
Inhibiting the b1 and b2 motoneurons alters the integral and proportional gains for pitch control, respectively. (**A**) Reconstruction of a fly experiencing and correcting for a pitch down mechanical perturbation. Walls show photomontages from three high-speed cameras. (**B**) PI controller model for rapid flight stabilization. In response to a disturbance, body angular velocity is measured via mechanosensory organs, subject to time delay, split into proportional and integral branches, and summed to determine corrective changes to wing kinematics. Blue and orange arrows highlight experimental finding that b1 and b2 motor unit inhibition attenuates integral and proportional feedback, respectively. (**C**) Example pitch down perturbations for a genetic control (left) (movie S11) and b1-silenced fly (right) (movie S12). Top: Change in pitch angle over time (blue traces). Bottom: Measured change in forward stroke angle over time (black dots), PI controller model fit (blue traces; 95% CI), P term (proportional; thin gray line), and I term (integral; dashed black line). The yellow bars indicate 7-ms magnetic pulse. (**D**) Summary statistics for PI controller model parameters (Eq. 1)—integral gain (*K*_i_; left), proportional gain (*K*_p_; center), and time delay (Δ*T*; right)—for b1-silenced flies (dark blue; *n* = 32) and two genetic controls (light blue and gray; *n* = 21 and 20). Upward and downward triangles represent distinct pitch up and down perturbation movies, respectively. Box plots show median (black line) and interquartile range. Lower case letters (i.e., “a” and “b”) indicate significance categories, determined via Kruskal-Wallis test with Bonferroni method multiple comparison (α = 0.05); a.u., arbitrary units. (**E**) Same as in (C) but with a genetic control (left) (movie S13) and b2-silenced fly (right) (movie S14). The integral term (dashed black line) is covered by the PI controller fit (solid orange line). (**F**) Same as (D) but with b2-silenced flies (dark orange; *n* = 17) and two genetic controls (light orange and gray; *n* = 22 and 22). See table S2 for full fly genotypes.

To directly test the contributions of these muscles to flight stabilization, we quantified responses to imposed midair perturbations from flies with chronically inhibited b1 and b2 activity. To conduct these experiments, we drove expression of the inwardly rectifying potassium channel Kir2.1 (*42*, *43*) in the b1 and b2 motoneurons (table S1 and Materials and Methods). Despite the kinematic responses observed in optogenetically b1-silenced flies (Fig. 2, D to F), chronic silencing of the b1 motoneuron did not preclude flight (fig. S3). To assay the effects of this chronic silencing on stabilization maneuvers, we imposed rapid, midair magnetic perturbations to freely flying flies with magnetic pins glued to their backs (Fig. 3A and fig. S4A). Using a custom tracking software (*44*), we extracted the flies’ corrective wing and body kinematics as they responded to either pitch or roll perturbations.

The observed kinematic changes under b1 or b2 inhibition become particularly transparent in the context of a PI controller framework (Fig. 3B) (*10*, *12*, *13*), which provides a reduced-order description for the corrective response. In the case of pitch perturbations (*11*, *12*), this PI model predicts changes in forward stroke amplitude (Δ_fwd_ϕ) as a function of time (*t*)

$$\Delta_{\mathrm{fwd}}\phi (t)=K_{p}{\dot{\theta }}_{b}(t-\Delta T)+K_{i}\Delta \theta_{b}(t-\Delta T)$$ (1)

where *K*_p_, *K*_i_, and Δ*T* are the proportional gain, integral gain, and time delay of the PI controller, respectively. Thus, the change in forward stroke angle (Δ_fwd_ϕ) at time *t* is quantitatively predicted by a linear combination of the body pitch angular displacement (Δθ_b_) and pitch velocity (${\dot{\theta }}_{b}$) at an earlier time point, *t* − Δ*T*. In this controller framework, the error signal (${\dot{\theta }}_{b}$) is assumed to be measured by the halteres, specialized mechanosensory organs that are thought to act similar to gyroscopes, measuring body angular velocities and providing the primary drive for fast flight control reflexes (*45*–*48*). Measurements of pitch angular displacement (Δθ_b_)—defined relative to the fly’s preperturbation attitude, i.e., Δθ_b_(*t*) = θ_b_(*t*) − θ_b_(0)—is then obtained via integration of the angular velocity signal. Note that this model could also be cast as a proportional-derivative (PD) controller, with angular displacement as the proportional term (P) and angular velocity as the derivative term (D); here, we use the nomenclature of a PI controller model to emphasize the presumed computation being performed on sensory information (i.e., angular velocity information from the halteres), consistent with previous studies (Supplementary Text) (*12*, *13*). The controller gain coefficients in Eq. 1, *K*_p_ and *K*_i_, determine the relative weights of angular velocity and displacement to the corrective response; the time delay, Δ*T*, corresponds to the reflex latency. Comparing these controller parameters in b1- and b2-silenced flies versus genetic controls allows for directly testing the roles of the b1 and b2 motor units in the reflex response.

We illustrate this strategy by comparing pitch perturbation events for individual flies: one from a genetic control group (Fig. 3C, left, and movie S11) and one from the b1-silenced group (Fig. 3C, right, and movie S12), both selected to illustrate the phenotypic differences observed in control strategies across genotypes. For similar maximum pitch deflections (−11.1° and −10.1° for the genetic control and the b1-silenced flies, respectively), the control group fly was able to return to its original orientation roughly 25 ms after the onset of the magnetic field pulse, while the b1-silenced fly leveled off at a pitch angle below its preperturbation orientation (Fig. 3C, top). In both cases, the PI controller model (Fig. 3C, bottom) quantitatively predicts the time course of forward stroke angle (Δ_fwd_ϕ). For the b1-silenced fly, however, the integral gain (*K*_i_) obtained from the fit is negative. This result is counterintuitive, as it would indicate a control law pushing the fly away from its initial orientation. Overall, b1-silenced flies showed a statistically significant decrease in the integral gain (*K*_i_) of the PI controller model as compared to genetic controls (Fig. 3D, left), whereas the proportional gain (*K*_p_) and time delay (Δ*T*) were not significantly different across genotypes (Fig. 3F, middle and right). Performing the same analysis on roll stabilization, we found no effect of b1 motoneuron silencing on feedback control (fig. S4). Together, these results indicate that b1 motoneuron silencing primarily affects pitch stabilization and does so in a manner that is captured by a single parameter in the PI controller model: the integral gain, *K*_i_ (Fig. 3B, blue arrow).

We applied the same strategy to elucidate the role of b2 in the stabilization reflexes (Fig. 3, E and F). Pitch perturbation events for a genetic control fly (Fig. 3E, left, and movie S13) and a b2-silenced fly (Fig. 3E, right, and movie S14)—again selected to highlight group-level differences—are both well fit by the PI controller model. Here, however, the PI controller fit to the corrective response of the b2-silenced fly lacks a proportional term, i.e., *K*_p_ = 0. This trend holds across flies: Compared to genetic controls, b2-silenced flies exhibited reduced proportional gain, whereas the distributions of other controller coefficients (*K*_i_ and Δ*T*) were not significantly different (Fig. 3F). We performed the same analyses using roll perturbations and found that b2 silencing had no measurable effect on roll stabilization (fig. S4). Thus, silencing the b2 motoneuron uniquely affected the proportional term, *K*_p_, for pitch control (Fig. 3B, orange arrow).

**Fig. 4.**
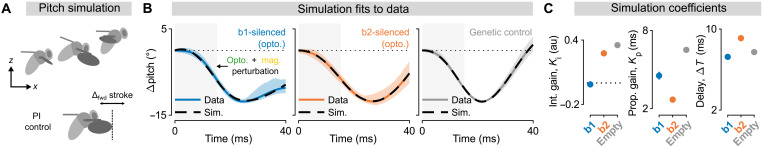
Simulating the effects of b1 and b2 silencing on pitch stabilization. (**A**) Illustration of time snapshots from longitudinal flight simulation (top) and change in forward stroke angle as a control parameter according to Eq. 1 (bottom). (**B**) Change in body pitch angle over time for simulated flies (dashed black lines) fit to experimental data (population average with 95% CI envelope). Columns correspond to different genotypes: *b1-GAL4* > *UAS-GtACR1* (“b1-silenced”; left; *n* = 41 movies), *b2-GAL4* > *UAS-GtACR1* (“b2-silenced”; middle; *n* = 32), and empty > *UAS-GtACR1* (“genetic control”; right; *n* = 62). The gray bars represent the 15 simultaneous LED and magnetic field stimuli, which optogenetically silence and impose external torque, respectively. (**C**) PI controller parameters from simulation fits in (B)—integral gain (*K*_i_; left), proportional gain (*K*_p_; middle), and time delay (Δ*T*; right)—for each genotype. Error bars show 95% CI. See table S2 for full fly genotypes.

This simple interpretation—b1 and b2 actuating integral and proportional control for pitch stabilization, respectively—is reinforced by matching simulations of flapping flight (*3*, *12*) to experimental data from optogenetically silenced flies undergoing magnetic perturbations (Fig. 4 and Supplementary Text). Here, we optimized the PI controller parameters in simulated flies to best reproduce the averaged pitch kinematics observed in real flies, grouped by genotype. To focus on the pitch degree of freedom, simulated flies were constrained to move in only two spatial (forward/back and up/down) and one rotational (pitch) dimensions (Fig. 4A) and were prescribed three-dimensional (3D) wing kinematics based on a simplified parameterization (see Materials and Methods) (*3*, *12*). Because of these simplifications and the explicit incorporation of the fly’s body dynamics, our simulations represented a distinct and complementary approach to the direct controller parameter fits in Fig. 3 (C to F). Using this approach, we quantitatively captured the changes in body pitch angle over time for flies undergoing simultaneous magnetic perturbation and optogenetic silencing (Fig. 4B). Consistent with our previous results, the controller parameters (*K*_i_, *K*_p_, and Δ*T*) obtained from these simulation fits show that b1 silencing reduces integral gain (Fig. 4C, left), while b2 silencing reduces proportional gain (Fig. 4C, middle).

## DISCUSSION

Our finding that the b1 and b2 motor units act as elemental control features in the pitch flight controller of *Drosophila* confirms a previous hypothesis that the two physiological categories of steering muscles—tonic (e.g., b1) and phasic (e.g., b2)—actuate integral and proportional control, respectively (*30*). On the basis of these results and previous studies showing a correspondence between anatomical groupings and their recruitment for maneuvers about different rotational axes (*29*, *30*), we conjecture that other tonic and phasic muscles in the wing motor system might be similarly mapped onto the integral and proportional controller parameters for yaw and roll. Moreover, while these connections between muscle physiology and behavioral function are particularly amenable to investigation in the specialized fly wing motor (*29*, *30*), we suspect that similar organizational principles generalize across animals: Functional stratification is a ubiquitous feature of muscle systems, with tonic and phasic muscle fibers types found not only in arthropods (*49*) but also throughout vertebrates (*50*, *51*).

Experimental approaches like the one demonstrated here will become more broadly applicable as genetic tools continue to proliferate. For instance, new driver line collections targeting sparse cell populations are actively being developed and will allow us to extend the methods used here beyond the motor system. Moreover, additional tools such as SPARC (**52**), which refine genetic expression patterns, will allow us to investigate cell types for which sufficiently sparse driver lines do not exist, as well as perform unilateral neuronal manipulations. These techniques will be especially useful for investigating circuitry upstream of the flight motor.

While our results illustrate the utility of combining top-down behavioral modeling in freely flying animals with bottom-up manipulations for probing neuromuscular systems, these approaches become even more powerful in the broader context of the field. Our investigations build on pioneering work studying the insect flight control system by allowing active manipulation of specific neurons in intact, untethered flies and providing the opportunity to explicitly test the functional role of specific neurons during naturalistic behavior. This approach can, in turn, guide investigations into subtler phenomena in tethered animals. For instance, while both chronic and optogenetic silencing of the b1 motoneuron produced strong phenotypic differences in control strategy, we are currently unable to record or manipulate the precise temporal phase of the b1 muscle in free flight despite phase being a factor known to influence wing kinematics (*32*, **53**). Electrophysiological studies in tethered flies (*31*, *32*, *34*), guided by the knowledge that the b1 motor unit actuates integral control, could allow for the temporally precise measurements and manipulations necessary to elucidate the role of b1 firing phase in the flight stabilization reflex. These synergies should facilitate a more complete understanding of how flight control is actuated. Last, when used alongside electron microscopy connectomics (**54**), which allow investigations into the relevant upstream sensory and interneuron circuitry, these approaches will likely provide critical insights into the full sensorimotor cascade for the PI controller and the neuromuscular underpinnings of flight control.

## MATERIALS AND METHODS

### Fly stocks and fly handling

Flies used for optogenetic experiments were reared in the dark at room temperature on 0.4 mM retinal food (Media Facility, HHMI Janelia Research Campus). Flies used for all other experiments (e.g., mechanical perturbation) were raised at room temperature on standard fly medium made from yeast, agar, and sucrose with a 12-hour light/12-hour dark cycle. Female flies, 3 to 6 days after eclosion, were used for all flight experiments. A full list of *Drosophila melanogaster* stocks used in this paper is given in table S1.

### Immunohistochemistry

Light microscopy images in Fig. 1 and figs. S1 and S5 were obtained using a protocol similar to the one described in (**55**). Briefly, full central nervous systems were dissected into PBS (phosphate-buffered saline) and fixed in 4% paraformaldehyde in PBS for 35 min at room temperature. Fixed tissues were then washed in PBT (PBS containing 0.1% Triton X-100) and incubated with primary antibodies diluted in PBT overnight at 4°C. The next day, samples were washed in PBT for several hours at room temperature and then incubated with secondary antibodies diluted in PBT overnight at 4°C. Samples were then washed all day with PBT, placed onto polylysine-coated coverslips, dehydrated through an ethanol series, cleared in xylenes, and mounted in DPX (Sigma-Aldrich). Adult central nervous system tissues were then imaged on a Leica SP6 confocal microscope with optical sections at 0.3-mm intervals. Maximum intensity projections (as shown in Fig. 1, B and C, and figs. S1, A to D, and S5, A and B) were generated using ImageJ.

Phalloidin images were obtained using a protocol similar to the one detailed in (*30*). Briefly, a razor blade was used to hemisect thoraces of adult female flies frozen in Tissue-Tek O.C.T. (Electron Microscopy Sciences, catalog no. 62550-01). Samples were then fixed in a solution of 4% paraformaldehyde in PBS for 45 min and subsequently washed three times in PBT. Primary antibodies and phalloidin stain were then added, and the samples were mutated for 7 to 10 days at 4°C. After the staining period, samples were rinsed in PBT and cleared using the SeeDB protocol (**56**). Samples were then mounted with SeeDB in between two glass coverslips, with another glass coverslip placed on top and clear nail polish used to seal the sample in. These samples were subsequently imaged using a Zeiss LSM 880 upright confocal microscope. The following stains/antibodies were used in the above protocols: rabbit polyclonal anti–green fluorescent protein (GFP) (Thermo Fisher Scientific, catalog no. A11122), rat anti–DN-cadherin (DSHB, DN-Ex #8), Alexa Fluor Plus 405 phalloidin (Invitrogen, catalog no. A30104), rabbit polyclonal anti-GFP (Torrey Pines, catalog no. TP401), and Alexa Fluor 488 goat anti-rabbit (Invitrogen, catalog no. A27034).

### Fly preparation

For perturbation experiments, individual flies were anesthetized at 0° to 4°C, at which point we carefully glued 1.5- to 2-mm-long, 0.15-mm-diameter ferromagnetic pins to their notum (dorsal thoracic surface). The pins were oriented to lie in the fly’s sagittal plane for pitch perturbation experiments; for roll experiments, the pins were oriented perpendicular the fly’s sagittal plane. Experiments with unpinned flies showed that the addition of the pin did not qualitatively alter flies’ flight kinematics. The attachment of pins adds mass that is comparable to natural intra-fly mass variation and adds negligibly to the off-diagonal components of the fly’s inertia tensor [for detailed calculations, see (*12*, *13*)].

### High-speed videography

We performed experiments with 15 to 30 flies prepared as above, all with the same genotype. We released these flies into a transparent cubic flight chamber with a side length of 13 cm. The center of the chamber was filmed by three orthogonal high-speed cameras (Phantom V7.1) at 8000 frames/s and 512 × 512 pixel resolution, with the three cameras sharing a mutual filming volume of ∼8 cm^3^ (Fig. 1D). Each camera was backlit by a focused 850- ± 30-nm near-infrared light-emitting diode (LED) (Osram Platinum Dragon). An optical trigger—created using split, expanded beams from a 5-mW, 633-nm HeNe laser (Thorlabs, HRR050) passed through a neutral density filter (Thorlabs, NE20A) with an optical density of 2.0 and incident upon two photodiodes (Thorlabs, FDS100)—was used to detect the entrance of flies into the filming volume of the high-speed cameras during experiments and initiate filming (*44*). Before each experiment, we calibrated the cameras using the easyWand system from (**57**).

### Optogenetic experiments

For each optogenetic experiment, 10 to 30 flies were released into the flight chamber described above for approximately 12 hours. To apply midair optogenetic stimulation, we used the optical trigger circuit described above to deliver a 50-ms bout of light stimulation from a collimated LED source placed outside the chamber (Fig. 1D) whenever a flying fly entered the center of the filming volume. This trigger also initiated filming with the high-speed cameras, which recorded flight activity before, during, and after the application of the light stimulus at 8000 frames/s. To apply the light stimulus, the optical trigger circuit drove a 50-ms duration voltage pulse to an LED driver (Thorlabs, LEDD1B), which was connected to either a 625-nm red LED (Thorlabs, M625L4) or a 565-nm green LED (Thorlabs, M565L3) for optogenetic excitation (CsChrimson) or inhibition (GtACR1) experiments, respectively. Both red and green LED sources were outfitted with a collimating attachment (Thorlabs, COP2-A) to generate a 50-mm-diameter beam profile. The cross-sectional area of this beam was large enough so that a fly anywhere in the filming volume of all three cameras would necessarily be hit by the light source, and the collimation ensured that the stimulus intensity was uniform regardless of the fly’s location within the filming volume.

Unless otherwise noted, the stimulation LEDs were driven with a 1-A current, resulting in intensities of 731 and 316 μW/mm^2^ for the red and green LEDs, respectively. Despite the optical trigger’s 633-nm HeNe laser ostensibly falling in the range of CsChrimson sensitivity, the optical filters on this light source ensured that the laser’s intensity, ∼0.16 μW/mm^2^, was two to three orders of magnitude lower than any applied LED stimulus. Moreover, we did not observe any changes to flight behavior resulting from the HeNe light when LED stimulation was withheld.

To prevent outside light contamination during these optogenetic experiments, the entire flight apparatus was surrounded by blackout curtains. Because flies are unlikely to initiate flight bouts in total darkness, a dim, blue fluorescent light bulb was used to illuminate the arena during experimental trials.

To analyze the video data from these optogenetic experiments, “pre-stim” and “stim” periods—as in Fig. 2 and fig. S2—were defined relative to the onset of the LED stimulus to capture data before the onset of optogenetic manipulation and during optogenetic manipulation, respectively. The four wingbeats preceding the onset of the LED stimulus, but not the one including it, were defined as the pre-stim period. The fourth to seventh wingbeats after the LED onset, with the LED still on, were defined as the stim period. The results of the optogenetic data analysis were not sensitive to the particular wingbeats selected for the stim period, and the selection of stim wingbeat numbers was based on visual inspection. Per-fly averaged wing kinematic data from pre-stim and stim periods were used both in plots of mean optogenetically evoked wing kinematics (Fig. 2, B and E, and fig. S2, A and B) and to calculate aerodynamic forces and torques using a quasi-steady aerodynamic model (Fig. 2, C and F, and fig. S2, C and D).

### Mechanical perturbation experiments

For each mechanical perturbation experiment, 10 to 20 flies were prepared by gluing small ferromagnetic pins to the dorsal side of their thoraces (see above) and subsequently released into the flight chamber. Similar to the optogenetic experiments described above, an optical trigger circuit was used to apply a variable duration magnetic field pulse whenever a flying fly entered the center of the filming volume. High-speed cameras were used to record flight activity before, during, and after the application of the magnetic field at 8000 frames/s.

The impulsive magnetic field was generated by the optical trigger supplying a rapid current pulse to a pair of Helmholtz coils mounted on the top and bottom faces of the flight chamber. Because of the positioning of the Helmholtz coils, this produced a roughly uniform vertical magnetic field in the center of the filming volume, triggered by the entrance of a fly entered into this region of the flight chamber. Typical magnetic field strengths were on the order of ∼10^−2^ T. The magnetic field from the coils acted on the magnetic moment of the ferromagnetic pin glued to the fly, in turn, generating a moment about either the fly’s pitch or roll axis, depending on the relative orientation of the field and pin (Fig. 1D, inset). Further details of this procedure are described in (*10*, *12*, *13*).

Most experiments using this method for imposing external magnetic torques were performed with chronically silenced flies (Fig. 3, C to F), with the magnetic field applied for 7 ms. However, this method could also be combined with the protocol for midair optogenetic manipulation described above to both optogenetically silence and mechanically perturb the same fly in a single movie. Combined optogenetic and mechanical perturbations were performed in two ways. In the first way, the LED and Helmholtz coils were powered simultaneously for 15 ms (Fig. 4). In the second way, the two signals were temporally offset and given different durations (fig. S6). Specifically, the LED was turned on for the time range *t* = 0 to 50 ms, while the magnetic field was applied for *t* = 15 to 22 ms, resulting in a 7-ms magnetic field pulse beginning 15 ms after the onset of the optogenetic LED (see the fig. S6 schematic).

### Flight data selection and kinematic extraction

Of the data collected in both optogenetic and mechanical perturbation experiments (as described above), we restricted our attention to videos that were amenable to kinematic analysis. Broadly, we required flight movies to contain the fly in the field of view of all three high-speed cameras long enough to analyze pre- and post-perturbation onset flight kinematics. In our temporal coordinates, the perturbation onset occurs at time *t* = 0 ms, so we required the fly to be visible from all three camera views in the range *t* ∈ [ − 10,30 ms] for a particular movie to merit analysis. For just mechanical perturbation experiments, we imposed a slightly stricter set of criteria in addition to this time limit. Namely, we required that the perturbation acts primarily along a single rotational axis and that there was no evidence of the fly performing a volitional maneuver before perturbation. Both of these criteria were imposed in an attempt to cleanly isolate corrective maneuvers for a single rotational degree of freedom.

To extract kinematic data from the three high-speed camera views, we used the custom-developed 3D hull reconstruction algorithm detailed in (*44*). Using this algorithm, we obtained a 12 degree-of-freedom description of the fly—the 3D position of the fly center of mass and the three full sets of Euler angles for the fly body, left wing, and right wing—at each time point. For time points in which occlusion precluded the direct extraction of a particular kinematic variable, we used a cubic spline interpolant to fill in missing data values. For most analyses, raw body kinematics were filtered using a 100-Hz low-pass filter. Raw wing kinematics were smoothed using the Savitzky-Golay method. For the wing stroke angle, we used a polynomial order seven with a window size of 21 frames (2.625 ms); for the wing deviation and rotation angles, we used a polynomial order five and a window size of 11 frames (1.375 ms). Figure S7 shows example wing and body Euler angles from the perturbation movies shown in Fig. 3 (C and E) (movies S11 to S14) as an illustration of this process of kinematic extraction.

To average wingbeat kinematics across flies—as in Fig. 2 (B and E) and fig. S2 (A and B)—we segmented wingbeats from time series data based on stroke angle maxima, i.e., the dorsal-most part of the wing stroke. Segmented wingbeat kinematics were then aligned to nondimensional wingbeat cycle time using a sixth-order Fourier series fit (MATLAB’s fit.m using the “fourier6” option) to evenly resample the Euler angle values. With all segmented wingbeats sampled according to a common nondimensional time, wing Euler angle traces could be directly averaged without incurring errors because of varying wingbeat frequency across flies/wingbeats.

### Controller model fitting for single-trial data

For each movie of a fly performing a corrective maneuver in response to a mechanical perturbation—as in Fig. 3 (C to F) and figs. S4, C to F; S8; and S6—we fit a PI model to the kinematic data obtained as above. The equations for pitch and roll PI controller models are given in Eq. 1 and eq. S1 and are based on previously derived models from (*10*, *12*, *13*). Note that the sign convention chosen for Eq. 1 and eq. S1 was selected so that the gain coefficients, *K*_i_ and *K*_p_, are assumed to be positive for stable systems, as in (*12*, *13*), largely consistent with the poles of the zero delay approximation to the characteristic polynomial (eq. S10).

To fit the controller coefficients for each perturbation movie, we performed a nonlinear least squares fit (Levenberg-Marquardt) for the gain coefficients, *K*_i_ and *K*_p_, along with a grid search for the time delay, Δ*T*. Thus, for each perturbation movie, we obtained fitted values for the three terms in the PI controller model: proportional gain (*K*_p_), integral gain (*K*_i_), and time delay (Δ*T*). Uncertainty in fit parameters was estimated using the fit parameter covariance matrix at the objective function minimum. The covariance matrix was approximated as *C* ≈ σ^2^(*J*^T^*J*)^−1^, where *C* is the covariance matrix, σ^2^ is the variance of the fit residuals, and *J* is the calculated Jacobian at the objective function minimum. The within-genotype spread in fitted controller parameters (e.g., in Fig. 3, D and F) was attributed to a combination of variation in morphology—both across individuals and due to dehydration over time—and the noise injected into the system by fitting time-domain models to relatively short-duration perturbation events.

Figure S9 shows additional example controller fits for motoneuron-silenced flies and genetic controls, as in Fig. 3 (C and E). These additional examples show data from perturbation events corresponding to representative values for the controller parameter affected in the experimental group (*K*_i_ for b1-silenced flies and the associated genetic controls, fig. S9A; *K*_p_ for b2-silenced flies and the associated genetic controls, fig. S9B).

### Fitting flight simulation parameters for averaged data

Our flapping flight simulation, similar to ones previously reported (*3*, *12*), poses the equations of motion for the fly in two translational (forward and vertical) and one rotational (pitch) degrees of freedom to study the dynamics of pitch stabilization in a reduced order framework. We used a set of analytic expressions (*3*) to prescribe the wing motion of simulated flies, with changes to the wing forward stroke angle implemented continuously on the basis of the PI controller scheme described above (Fig. 3 and Eq. 1), which allowed us to calculate instantaneous aerodynamic forces and torques acting on the wings using a quasi-steady model (*39*, **58**). To mimic the details of our experiment, we included an option to impose an external pitch torque of tunable magnitude and duration of the simulated fly. For each simulation run, we solved the equations of motion for the fly using MATLAB’s delayed differential equation solver, dde23.m. An expanded description of the flight simulation can be found in the Supplementary Text (“Flight simulation” section).

When fitting simulation results to averaged experimental data, we held constant all parameters but the three PI controller parameters (*K*_i_, *K*_p_, and Δ*T*) and the strength of the perturbing pitch torque. The cost function minimized in each fit was the least squares difference between the simulated and measured body pitch angle in a 40-ms window beginning at the onset of the perturbation. We performed this nonlinear least squares fit using the Levenberg-Marquardt algorithm in MATLAB’s lsqnonlin.m function, with ≥12 randomized start points to avoid the solver getting trapped in local minima. To characterize the uncertainty in the final fit parameters, we estimated 95% confidence intervals for each fit parameter using the procedure described above for controller model fits to data.

For the simulation fits shown in Fig. 4, we used data used from experiments in which flies expressing the optogenetic silencer GtACR1 under different GAL4 driver lines—*b1-GAL4*, *b2-GAL4*, and *SS01062-GAL4*—were subjected to simultaneous LED and magnetic field pulses lasting 15 ms, thereby transiently inhibiting the targeted neurons and applying a mechanical perturbation. To lessen the influence of motion/rotation about degrees of freedom other than pitch, we performed simulation fits to genotype-averaged data, thereby generating a single set of simulated controller coefficients per genotype. Because the strength of the mechanical perturbation in our behavioral experiments varies depending on pin length, magnetization, and orientation, we first normalized the body pitch traces from each individual movie so that each time series had identical maximum pitch deflections. We then averaged these normalized traces and rescaled the resulting average to have maximum pitch deflection equal to the median perturbation amplitude across all movies (∼12°). This ensured that the population-averaged traces for each genotype had matching perturbation magnitudes.

### LexA/Gal4 intersectional strategy

To account for the off-target expression in the brain of *b1-GAL4* flies (fig. S5A), we used an intersectional approach to restrict Kir2.1 expression to the ventral nerve cord (VNC) in the b1 motoneuron chronic silencing experiments presented in Fig. 3 (C and D). In this approach, akin to the one used in (*43*), a Flp recombinase was driven by tshLexA (which expresses LexA in most neurons of the VNC) and used to excise a transcriptional stop cassette from a *10XUAS-Kir2.1* transgene, which, in turn, was driven by *b1-GAL4* (see table S1).

As a control for the presence of additional transgenes introduced in this intersectional approach, we performed a set of pitch and roll perturbation experiments using *5XUAS-Kir2.1* crossed to *b1-GAL4* flies lacking the LexA/LexAop transgenes, a cross that mirrors the experiments in Fig. 3 (E and F) using *5XUAS-Kir2.1* flies crossed to *b2-GAL4* flies. The results of these experiments, presented in fig. S8, were consistent with those reported in the main text.

### Calcium imaging

To image calcium activity in steering muscles, we tethered flies in an upright orientation similar to free flight and mounted them in the custom imaging rig described in (*30*). A 470-nm LED (Thorlabs) was used as an excitation light source and passed through a Chroma filter cube with a 480/40-nm excitation filter and 510-nm long-pass dichroic. GCaMP6f fluorescence was collected through a 10× 0.45 numerical aperture lens and 535/50-nm emission filter. Stroke amplitude and wingbeat frequency were simultaneously monitored during fluorescence imaging: the former using a camera (Basilar) and infrared light and the latter with a photodiode-based wingbeat analyzer.

We used the machine vision system Kinefly (**59**) to extract stroke amplitude in real time, which we, in turn, used as feedback for closed-loop control of visual stimulus. These visual stimuli were displayed to the fly using a cylindrical panoramic display screen made from LED panels, with 470-nm peak wavelength, as in (*30*). Using this, we presented the fly with both open-loop visual displays simulating rotation about the yaw, pitch, and roll axes, as well as closed-loop stripe fixation patterns.

From images of GCaMP6f fluorescence, we used the method of demixing described in (*30*) to extract signals from individual muscles. Briefly, this involved fitting measured signals to a generative model for muscle fluorescence based on anatomical priors and properties of the imaging apparatus. Because many of the muscles do not produce appreciable GCaMP6f signals during quiescence, we calculated the change in fluorescence, Δ*F*/*F*, with a baseline (*F*) determined by the first percentile of fluorescence signal on a per fly and trial basis.

## Supplementary Materials

### This PDF file includes:

Supplementary Text Figs. S1 to S10 Tables S1 to S4 References
