# Supplementary material for: Neuromuscular embodiment of feedback control elements in Drosophila flight
Source: Sci Adv. 2022 Dec 14;8(50):eabo7461. doi: 10.1126/sciadv.abo7461 (PMC9750141; doi:10.1126/sciadv.abo7461)
Supplement: Supplementary file 1 — Supplementary Text Figs. S1 to S10 Tables S1 to S4 References [file sciadv.abo7461_sm.pdf]

Science Advances
AAAS

Supplementary Materials for

Neuromuscular embodiment of feedback control elements in Drosophila flight

Samuel C. Whitehead *et al.*

Corresponding author: Samuel C. Whitehead, scw97@cornell.edu

Sci. Adv. **8**, eabo7461 (2022)
DOI: 10.1126/sciadv.abo7461

The PDF file includes:

Supplementary Text
Figs. S1 to S10
Tables S1 to S4
Legends for movies S1 to S14
References

Other Supplementary Material for this manuscript includes the following:

Movies S1 to S14

Supplementary Text

Functional recording from steering muscles

To confirm that silencing the b1 motoneuron inhibited the activity of the b1 muscle, we directly imaged the calcium activity in flight steering muscles using a previously described experimental paradigm (see Materials and Methods) (*30*). For these experiments, we co-expressed both i) the genetically encoded calcium indicator GCaMP6f in the wing steering muscles using the driver line *R39E01-LexA* and ii) Kir2.1 in the b1 motoneuron using the *b1-GAL4* driver line. We presented tethered flies with visual stimuli on an LED screen while simultaneously monitoring activity in steering muscles, along with wingbeat amplitude and frequency (Fig. S3A). Figure S3B shows examples of pixel-wise variance across entire $\sim$200 s bouts of closed-loop stripe fixation for one b1-silenced fly and two genetic control flies. While the genetic control examples show large pixel variance in the b1 muscle area, indicating that these flies modulated activity in the b1 muscle throughout the flight bout, this variance is not present in the b1-silenced flies.

At the population level, we analyzed both the wing kinematics and the activity of the imaged steering muscles during initiation and cessation of flight during bouts of closed-loop stripe fixation (Fig. S3C). Previous studies have demonstrated that the onset of flight is accompanied by a steep increase in activity across all steering muscles (*30*). We observed this increase in activity across all muscles in both genetic control lines; however, as expected, the b1-silenced flies exhibited significantly reduced activity in the b1 muscle during initiation. The lack of b1 activity in b1-silenced flies is also present at flight cessation, when many of the direct steering muscles show a peak in activity before entering quiescence. Interestingly, in Figure S3C b1-silenced flies show an increased level of hg4 activity relative to genetic controls; however, activity in the smaller hg (fourth axillary) muscles is more difficult to de-mix than in the larger muscles like b1 and b2, so it is unclear if this represents a real effect.

From these experiments, we conclude that chronic silencing of the b1 motoneuron using Kir2.1 effectively inhibits the activity of the b1 muscle.

Roll control

In parallel to the pitch perturbation experiments shown in Figure 3, we assayed flies' response to roll perturbations by applying brief (7 ms) magnetic pulses to flies with ferromagnetic pins glued to their dorsal thoracic surface, oriented normal to their sagittal plane. Experiments were carried out as described in Materials and Methods, with flies glued with pins for pitch and roll released simultaneously into the flight chamber. Figure S4A shows an example of a roll perturbation event, with the 3D model fly in the center showing the measured kinematics at five snapshots in time, and the photomontages from the three high-speed videos projected onto the walls of the illustration.

As described in previous studies (*13*), flies control for roll perturbations by modulating the difference in stroke amplitude between their left and right wings to produce counter torques about their roll axis. This left/right stroke amplitude difference ($\Delta_{\mathrm{LR}}\Phi$) modulation over time (t) is well-described by a time-delayed PI controller model (Fig. S4B) (*13*), which can be written:

$$\Delta_{\mathrm{LR}}\Phi(t) = K_{\mathrm{p}}\dot{\rho}(t-\Delta T) + K_{\mathrm{i}}\Delta\rho(t-\Delta T)\,, \tag{S1}$$

where, as in Equation 1, the controller parameters K_p, K_i, and ΔT correspond to the proportional gain, integral gain, and time delay, respectively. The variables ρ and $\dot{\rho}$ refer to the body roll angle and roll angular velocity, respectively. For each roll perturbation event, we extracted the body and wing kinematics from 3D reconstructions of the video (Materials and Methods) and fit for the control parameters K_p, K_i, and ΔT.

Figure S4C shows example roll perturbation events for a genetic control (left) and b1-silenced fly (right). Both flies experienced a rightward roll deflection (top) during the period when the magnetic field was applied, indicated by a yellow bar in the time series plots. The bottom row of Figure S4C shows the PI controller fits for these two flies (blue curves) along with the measured difference in left/right stroke amplitude (black dots). At the population level, we compared PI controller model coefficients (K_i, K_p, ΔT) between b1-silenced flies and genetic controls for roll control (Fig. S4D). We did not observe any statistically significant differences between the three tested genotypes for any of the control parameters. We also did not observe any significant differences between b2-silenced flies and genetic controls (Fig. S4E,F).

Determining the effects of b1 and b2 silencing on roll control is complicated by the fact that—unlike the case of pitch stabilization—corrective roll maneuvers require an asymmetric response in the left and right wings, while our motoneuron silencing using split-GAL4 driver lines is bilateral. However, previous imaging studies have shown that when presented with fictive roll stimuli, the change in activity of the b1 and b2 muscles is also left/right asymmetric, with activity increasing on the side of increased stroke amplitude, and vice versa (*30*). Thus, even with bilateral driver lines, we would expect to see changes in the response to roll perturbations in motoneuron-silenced flies. That we do not see changes to the roll response indicates that other muscle groups (e.g. the first and third axillaries (*29, 30*)) likely play a more important role than the basalars in the execution of roll control.

Quasi-steady aerodynamic model

To estimate the aerodynamic forces and torques produced by the wingbeat patterns observed in our experiments, we used a quasi-steady aerodynamic model developed in previous studies and validated using scaled mechanical models (*39, 58, 60*). Specifically, we calculated the total instantaneous quasi-steady aerodynamic forces on a wing, which can be separated into two terms: the translational ($\mathbf{F}_\mathrm{t}$) and rotational ($\mathbf{F}_\mathrm{rot}$) forces (*39, 58, 60, 73*). Following (*61*), we do not include the contribution of added mass in this model, as analytic expressions for added mass force contributions (*58*) are inaccurate in cases of large acceleration. Thus, the total quasi-steady aerodynamic force can be written:

$$\begin{aligned}
\mathbf{F} &= \mathbf{F}_\mathrm{t} + \mathbf{F}_\mathrm{rot}\,, \\
&= \left(F_\mathrm{L}\hat{\mathbf{l}} + F_\mathrm{D}\hat{\mathbf{d}}\right) + F_\mathrm{rot}\,, \\
&= \frac{1}{2}\rho S U_\mathrm{t}^2 \hat{r}_2^2(S)\left(C_\mathrm{L}(\alpha)\hat{\mathbf{l}} + C_\mathrm{D}(\alpha)\hat{\mathbf{d}}\right) + \left(C_\mathrm{rot}\rho U_\mathrm{t}\omega_x \bar{c}^2 R\int_0^1 \hat{r}\hat{c}^2(\hat{r})\,\mathrm{d}\hat{r}\right)\hat{\mathbf{n}}\,,
\end{aligned} \tag{S2}$$

where ρ is the air density; S the wing area; U_t the velocity of the wing tip; $\hat{r}_2^2(S)$ the non-dimensionalized second moment of wing area (*62*); α the wing angle of attack; C_L and C_D the lift and drag coefficients; C_rot the rotational force coefficient; ω_x the angular velocity of the wing about its spanwise axis; $\bar{c}$ the mean wing chord length; R the wing span length; $\hat{r}$ and $\hat{c}$ the non-dimensionalized span and chord; and $\hat{\mathbf{l}}$, $\hat{\mathbf{d}}$, and $\hat{\mathbf{n}}$ are unit vectors in the directions of the lift, drag, and wing surface normal. We used constant values for the morphological and

physical parameters in Equation S2 across flies; the values of these parameters are given in Table S4. The lift and drag coefficients (C_{L} and C_{D}) can be written as functions of the wing angle of attack (α) (*63*):

$$
\begin{aligned}
C_{\mathrm{L}}(\alpha) &= C_{\mathrm{L_{max}}} \sin(2\alpha) \\
C_{\mathrm{D}}(\alpha) &= \left(\frac{C_{\mathrm{D_{max}}} + C_{\mathrm{D_0}}}{2}\right) - \left(\frac{C_{\mathrm{D_{max}}} - C_{\mathrm{D_0}}}{2}\right) \cos(2\alpha)\,,
\end{aligned}
\tag{S3}
$$

where $C_{\mathrm{L_{max}}}$, $C_{\mathrm{D_{max}}}$, and $C_{\mathrm{D_0}}$ are dimensionless constants best fit for *Drosophila* flight (see Table S4).

To calculate the aerodynamic torque, $\mathbf{T}$, produced by a flapping wing, we use the approximation that the wing center of pressure is located 70% along the length of the wing span (*64*), and write:

$$\mathbf{T} = (0.7R\hat{\mathbf{r}} + \mathbf{r}_{\mathrm{hinge}}) \times \mathbf{F}\,, \tag{S4}$$

where, as above, R is the wing span length, $\hat{\mathbf{r}}$ is the wing span unit vector, $\mathbf{F}$ is the total aerodynamic force produced by the wing, and $\mathbf{r}_{\mathrm{hinge}}$ is the vector from the fly center of mass to the wing hinge (see Table S4). Because Equation S4 is written in the body-tied frame of reference, the pitching torque, T_{pitch}, corresponds to the y component of T. As we are primarily interested in the pitch torque, T_{pitch}, we write the hinge vector, $\mathbf{r}_{\mathrm{hinge}}$, as purely along the fly's long body axis ($\hat{x}_{\mathrm{body}}$), i.e. $\mathbf{r}_{\mathrm{hinge}} = r_{\mathrm{hinge}}\hat{x}_{\mathrm{body}}$. When presenting aerodynamic force calculations (Fig. S2), we normalize these values to the weight of the fly, i.e. $\mathbf{F} \rightarrow \mathbf{F}/mg$. Similarly, we normalize aerodynamic torque by the product of the fly's weight and wing span length, i.e. $\mathbf{T} \rightarrow \mathbf{T}/mgR$ (Fig. 2, S2).

Flight simulation

To simulate flapping flight, we used a framework similar to the one described in (*12*). In these simulations, we numerically solve the Newton-Euler equations of motion in the longitudinal plane, i.e. we restrict a simulated fly's motion to two translational and one rotational degrees of freedom, so that the state vector describing the fly's body, s, can be written:

$$\mathbf{s} = \left(x, \dot{x}, z, \dot{z}, \theta_{\mathrm{b}}, \dot{\theta}_{\mathrm{b}}\right)\,, \tag{S5}$$

where x and z denote the body center of mass coordinates in the forward and vertical directions, and θ_{b} the body rotation angle about the pitch axis. The dot notation (e.g. $\dot{x}$) refers to the derivative with respect to time of the given variable. In these coordinates, we write the Newton-Euler equations for rigid body motion in the fly body frame:

$$
\begin{aligned}
\ddot{x} &= \frac{F_x}{m} - g\sin\theta_{\mathrm{b}} + \dot{\theta}_{\mathrm{b}}\dot{z}\,, \\
\ddot{z} &= \frac{F_z}{m} - g\cos\theta_{\mathrm{b}} - \dot{\theta}_{\mathrm{b}}\dot{x}\,, \\
\ddot{\theta}_{\mathrm{b}} &= -\frac{1}{I_{\mathrm{pitch}}}\left(T_{\mathrm{pitch}} + C_{\mathrm{friction}}\dot{\theta}_{\mathrm{b}}\right) + B\cos(\theta_{\mathrm{b}} - \theta_{\mathrm{pin}})\left(H(t) - H(t - T_{\mathrm{pulse}}\right)\,,
\end{aligned}
\tag{S6}
$$

where m is the fly body mass; I_{pitch} is the pitch moment of inertia; g is the gravitational acceleration; F_x and F_z are the x and z components of aerodynamic forces generated by the flapping wings; T_{pitch} is the component

of aerodynamic torque generated by the wings; and C_{friction} is the coefficient of pitch rotational drag. The last term on the right hand side of the equation for $\ddot{\theta}_{\text{b}}$ is the simulation equivalent of the magnetic perturbation applied in our experiments (Fig. 3): H represents the Heaviside step function, so this term is nonzero only during the duration of the applied perturbation, $t \in [0, T_{\text{pulse}}]$. The angle that the magnetic pin makes with the fly body in the sagittal plane, θ_{pin}, is kept fixed throughout, while the magnetic perturbation strength, B, is kept constant for a given simulation run, but varied when the simulation is fit to data. Table S4 gives a list of the constant parameters used in our simulations.

To calculate the aerodynamic forces and torques generated by the flapping wings, we prescribed the wing kinematics of the simulated fly using a set of simplified equations as in (*3, 12*), and used a quasi-steady aerodynamic model to determine the forces generated by these flapping patterns (see Eq. S2 above). The parameterized form for the wing kinematics is given by:

$$\begin{aligned} \phi(t) &= \phi_0 + \phi_m \frac{\arcsin(K \sin(2\pi f t))}{\arcsin(K)} \,, \\ \theta(t) &= \theta_0 + \theta_m \cos(2\pi f t + \delta_\theta) \,, \\ \eta(t) &= \eta_0 + \eta_m \frac{\tanh(C \sin(2\pi f t + \delta_\eta))}{\tanh(C)} \,, \end{aligned} \tag{S7}$$

where $\phi(t)$, $\theta(t)$, and $\eta(t)$ are the wing Euler angles—stroke, deviation, and rotation (see Fig. 1F)—as a function of time; ϕ_0, θ_0, and η_0 are angle offsets; ϕ_m, θ_m, and η_m are amplitudes; δ_θ and δ_η are phase offsets; K and C are dimensionless parameters that tune the shape of the waveform; and f is the wingbeat frequency. For our flight simulations, all parameters but ϕ_0 and ϕ_m, which are used to set the forward stroke amplitude, were kept fixed and based on an optimization for hovering stability within our simulation framework, with the deviation angle, θ, set to 0° for simplicity and to focus on the effects of forward stroke angle modulation, as in (*3, 12, 65*). See Table S4 for the values of these parameters. For a given set of wing Euler angles, we write the unit vectors pointing in the direction of the wing span and chord ($\hat{\mathbf{r}}$ and $\hat{\mathbf{c}}$, respectively)—used to determine wingtip velocity, torque lever arm, etc. in Equations S2, S4—as:

$$\begin{aligned} \hat{\mathbf{r}} &= (\cos(\phi)\cos(\theta),\ \sin(\phi)\cos(\theta),\ \sin(\theta)) \,, \\ \hat{\mathbf{c}} &= (\sin(\eta)\sin(\theta)\cos(\phi) - \cos(\eta)\sin(\phi),\ \sin(\eta)\sin(\theta)\sin(\phi) + \cos(\eta)\cos(\phi),\ \cos(\theta)\sin(\eta)) \end{aligned} \tag{S8}$$

The simulated fly implements PI control for its pitch degree of freedom as in Equation 1, which computes the change in forward stroke angle, $\Delta_{\text{fwd}}\phi$. In terms of the wing kinematic parameters in S7, this corresponds to an added change to the stroke angle parameters ϕ_0 and ϕ_m given by:

$$\begin{aligned} \Delta\phi_0 &= \frac{1}{2}\Delta_{\text{fwd}}\phi \,, \\ \Delta\phi_m &= -\frac{1}{2}\Delta_{\text{fwd}}\phi \end{aligned} \tag{S9}$$

Because Equation 1 is defined for continuous time, we modify the time delay so that the value of $\Delta_{\text{fwd}}\phi$ is updated only once per wingstroke.

Using this simulation framework, we performed least-squares fits to experimental data, allowing the controller parameters (K_{i}, K_{p}, and ΔT) and the magnetic perturbation strength (B) to vary, and minimizing the difference between measured and simulated body kinematics (see Materials and Methods).

Simplified closed-loop model

Mechanical dynamics are crucial to locomotor control (*1, 66, 67*), so we further assessed the closed-loop dynamics of *Drosophila* pitch control in the presence of motoneuron manipulations by estimating a linearized plant model, and used the result to examine the closed-loop dynamics. Beginning with the equation of motion for the pitch degree of freedom (Eq. S6), we consider the unforced ($B = 0$) dynamics. We approximate the torque, T_{pitch}, as a linear combination of the delayed PI feedback $\Delta_{\text{fwd}}\phi$ (Eq. 1) and body pitch angle. This gives:

$$I_{\text{pitch}}\ddot{\theta}_{\text{b}}(t) + C_{\text{friction}}\dot{\theta}_{\text{b}}(t) + K\Delta\theta_{\text{b}}(t) = A\Delta_{\text{fwd}}\phi(t) \; , \tag{S10}$$

where $\Delta_{\text{fwd}}\phi(t)$ is given by the PI controller output (Eq. 1). The first fitting parameter, K, is analogous to a spring constant and captures the relationship between body pitch angle and torque that can arise, for example, due to an offset between the center of lift and center of mass (*11, 18*). The second fitting parameter, A, captures the scaling that relates changes in wing kinematic output to changes in pitch torque. The remaining terms in Equation S10 are defined as above: I_{pitch} and C_{friction} are the moment of inertia and coefficient of rotational drag about the pitch axis, respectively (Tab. S4); θ_{b}, $\dot{\theta}_{\text{b}}$, and $\ddot{\theta}_{\text{b}}$ are the body pitch angle, velocity, and acceleration.

Using Equation S10, measured body pitch kinematics, fitted controller model parameters, and literature values for I_{pitch} and C_{friction} (*68*), we performed a least-squares fit for the spring constant, K, and scaling term, A. With the resultant fit parameters (K=7.25 × 10^{-10} N m, A=–8.28 × 10^{-9} N m), the plant and controller transfer functions in our linearized system—P and C, respectively—can be written:

$$\begin{aligned} P(s) &= \frac{As}{I_{\text{pitch}}s^2 + C_{\text{friction}}s + K} \, , \\ C(s) &= \left(K_{\text{p}} + K_{\text{i}}/s\right) e^{-\Delta T s} \, , \end{aligned} \tag{S11}$$

where s is the Laplace transform complex frequency and $e^{-\Delta T s}$ represents the controller time delay. Here, $P(s)$ is taken to be the transfer function from $\Delta_{\text{fwd}}\phi(t)$ to $\dot{\theta}_{\text{b}}(t)$. Using MATLAB's `feedback` command, we generated the closed-loop transfer function $G(s) = P(s)/(1 - P(s)C(s))$, eliminating the pole–zero cancellation at $s = 0$ using MATLAB's `minreal`. Note that $G(s)$ maps torque perturbations to pitch angular velocities. We used `lsim` with G to simulate responses to a simulated 15 ms magnetic pulse, and integrated the result using MATLAB's `cumtrapz` command to calculate $\Delta\theta_{\text{b}}(t)$. We tuned the impulse magnitude, i.e. the magnetic field strength B, by hand until the output of the linearized dynamics had approximately the same amplitude as the data and nonlinear simulation. We found that the resulting value of B matched the range of values obtained for B in our nonlinear fitting (note that the author who tuned B was blind to the range of values fitted in the nonlinear simulations). The time course of simulated linearized dynamics showed excellent qualitative agreement with both our behavioral data and flapping flight simulations (Fig. S10A).

The accuracy with which this linearized model captured our experimentally observed dynamics allowed us to further explore the effects of differing control strategies on stability. In particular, using Equation S10, we calculated the damping ratio, ζ, for each fly genotype (Fig. S10B), under a simplifying assumption of no time delay in the feedback loop. As shown in Figure S10B, the closed-loop dynamics for genetic control flies were slightly underdamped ($\zeta \approx 0.63$). For b1-silenced flies, the dynamics were slightly overdamped ($\zeta > 1$), resulting in a slower system response. For b2-silenced flies, the system became sufficiently underdamped to go unstable when the time delay was reintroduced, although only marginally so, resulting in a very slow unstable mode. Estimating open-loop transfer functions can sensitively depend on the closed-loop dynamics (*25*), and

we suspect that this instability may be an artifact of the relatively short time window over which we measured kinematic data, thus fitting short-term transients while not capturing steady-state stability (we made no effort to constrain the parameter estimates to ensure stability). This linear analysis highlights the critical role of velocity feedback (*69*) (i.e. analogous to a traditional "derivative controller," since the mechanosensors produce delayed angular-velocity-dependent feedback (*70, 71*)) on pitch stability in addition to the importance of integral control of pitch velocity (i.e. "proportional" to change in body-pitch angle) on transient performance.

Motoneuron driver lines

Because the *b1-GAL4* (*MB258C-GAL4*) driver line contained off-target VNC expression in the neck tectulum and abdominal neuromere (*72*), we performed optogenetic excitation and inhibition experiments as in Figure 2 using the split-GAL4 line *SS04528-GAL4*, which targets the b1 motoneuron but does not share common off-target expression with *b1-GAL4* (*40*). Figure S5 shows the expression pattern of *SS04528-GAL4* in the brain and VNC (c.f. Fig. S1A,B), as well as the body and wing kinematic changes evoked via optogenetic excitation (Fig. S5C–E) and silencing (Fig. S5F–G) compared to both *b1-GAL4* and the empty split *SS01062-GAL4*. The body and wing responses to both excitation and silencing between *SS04528-GAL4* and *b1-GAL4*—light and dark blue in Figure S5, respectively—are in agreement. We observed the same phenotypic similarity when comparing the full set of wing Euler angles for wingbeats prior to and during the LED stimulus (Fig. S2). Taken together, these results indicate that the flight phenotypes we observed using *b1-GAL4* are indeed due to manipulation of the b1 motoneuron, and not off-target cells. We were unable to find an alternate driver line targeting the b2 motoneuron to perform complementary tests of *b2-GAL4*; however, *b2-GAL4*'s relatively low level of off-target expression (Fig. 1C, S1C,D) and the agreement between our results (Fig. 2, S2) and previous electrophysiology/imaging studies (*30–32*) suggest that the phenotypes we observed are very likely due to b2 motoneuron manipulation.

Combined optogenetic and mechanical perturbation

As an alternative to chronic silencing, we performed experiments in which we crossed our motoneuron driver lines to *UAS-GtACR1* in order to apply combined optogenetic silencing and magnetic perturbations. We performed two versions of these experiments. In the first, both LED and magnetic field were applied simultaneously for 15 ms, as in the case of the data used to fit for simulation parameters in Figure 4B,C. In the second version, the two signals differed in both their duration and onset time—specifically, we used a 50 ms LED pulse for optogenetic silencing and a 7 ms magnetic field pulse that began 15 ms after the onset of the LED pulse. For this latter paradigm, we performed fit PI controller models to the pitch and roll stabilization responses (Fig. S6), as in the analysis of chronic silencing data. Again, our findings using this combined optogenetic and magnetic perturbation were consistent with the results of the chronic silencing experiments, only with a smaller effect size: b1- and b2-silenced flies showed decreases integral and proportional gains for pitch control, respectively (Fig. S6A), and we observed no effect on roll control (Fig. S6B).

Controller model nomenclature

As alluded to under Equation 1, the proportional-integral (PI) controller reported in this paper (Eq. 1, S1) would instead be an instance of proportional-derivative (PD) control if the sensor was presumed to measure positional error directly. In that case, the derivative (D) control term would be obtained by differentiation of this position signal, and correspond to angular velocity. Thus, because Equations 1 and S1 contain an angular position term and its derivative (angular velocity), they appear similar to a traditional PD control scheme for a mechanical system. Crucially, however, the control scheme instantiated by flies for rapid flight stabilization is thought to be based on *angular velocity*: a significant body of previous research indicates that the halteres—specialized mechanosensory organs unique to flies—measure body angular velocities and provide the primary sensory input for the fast flight control reflexes (*45–48*). Our use of the "PI" label is therefore the appropriate nomenclature in this context, as the computation being performed for rapid flight stabilization is truly proportional and integral control on the sensory output.

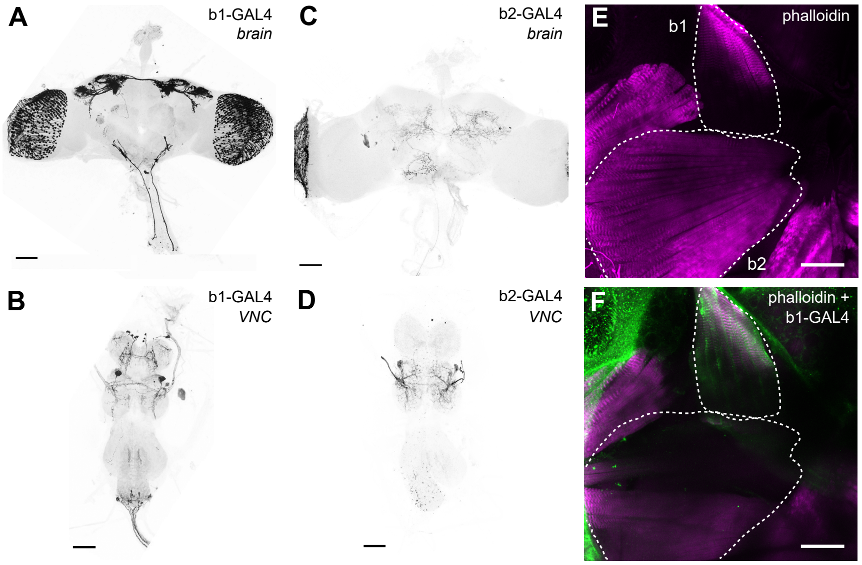

Figure S1: Maximum intensity projection (MIP) images of the split driver lines *b1-GAL4* and *b2-GAL4*. **A** Maximum intensity projection (MIP) brain image from a *b1-GAL4* > *CsChrimson* fly. Black corresponds to mVenus, light gray to DNCad (neuropil). **B** MIP VNC image from a *b1-GAL4* > *CsChrimson* fly (same image as in Figure 1B). **C**,**D** Same as **A**,**B** but with *b2-GAL4* > *CsChrimson* flies. **D** shows the same image as in Figure 1C. **E**,**F** Phalloidin-stained thoracic hemisection from a *UAS-GFP* > *b1-GAL4* fly showing wing musculature. b1 and b2 muscles outlined with white dashed lines. **E** shows just the phalloidin channel (magenta) across the full stack; **F** shows the MIP of a partial stack with both phalloidin and GFP (green) expression innervating the b1 muscle. All scale bars 50 µm. See Table S3 for full fly genotypes.

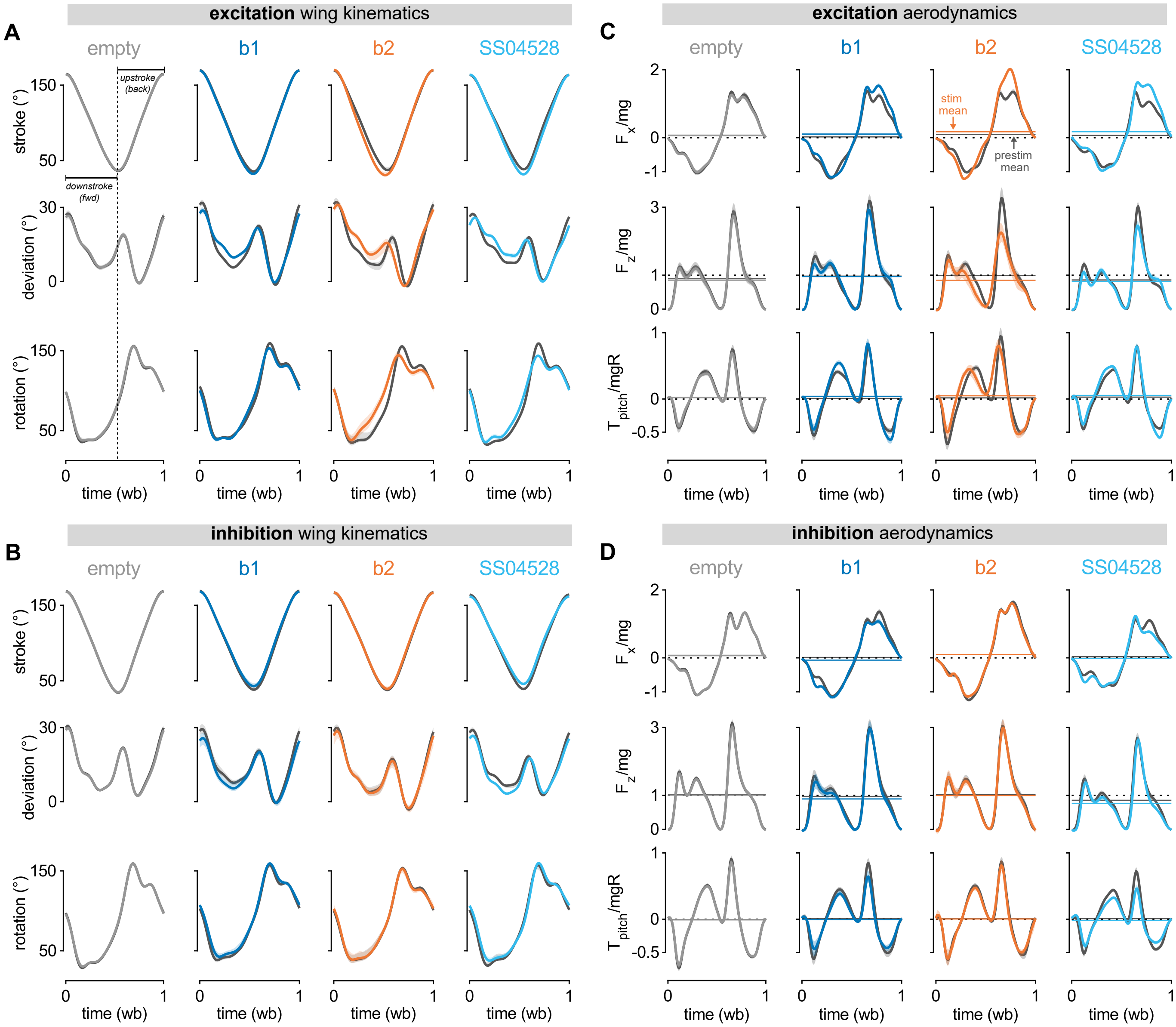
A
excitation wing kinematics
empty
b1
b2
SS04528
stroke (°)
150
50
upstroke (back)
downstroke (fwd)
deviation (°)
30
0
rotation (°)
150
50
time (wb)
0
1
C
excitation aerodynamics
F_x/mg
F_z/mg
T_{pitch}/mgR
stim mean
prestim mean
2
0
-1
3
1
0
1
0
-0.5
B
inhibition wing kinematics
D
inhibition aerodynamics

Figure S2: Extended results on the optogenetic activation and silencing of the b1 and b2 motoneurons. **A** Population-averaged time courses of the three wing Euler angles (stroke, deviation, and rotation), averaged across the left and right wings, comparing pre- (dark gray) and during-LED-stimulus (light gray, blue, orange) across a single wingbeat for bouts of optogenetic excitation with CsChrimson. Columns from left to right: *empty* (aka *SS01062-GAL4*; light gray), *b1-GAL4* (dark blue), *b2-GAL4* (orange), and *SS04528-GAL4* (light blue/cyan; targets b1 motoneuron). **B** Same as in **A** but for bouts of optogenetic silencing with GtACR1. **C** Population-averaged time courses of normalized quasi-steady aerodynamic forces and torques, summed across the left and right wings, comparing pre- (dark gray) and during-LED-stimulus (light gray, dark blue, orange, light blue/cyan) across a single wingbeat for bouts of optogenetic excitation with CsChrimson. Rows from top to bottom: forward/backward force (F_x/mg), up/down force (F_z/mg), and pitch torque (T_{pitch}/mgR). Columns correspond to the empty, b1, b2, and SS04528 genotypes as in **A**. Solid horizontal lines show values of force or torque obtained by averaging the plotted time series over the full wingbeat. Dotted horizontal lines show the expected value of normalized force or torque for steady, hovering flight: $F_x/mg = 0$ (top row), $F_z/mg = 1$ (middle row), and $T_{\text{pitch}}/mgR = 0$ (bottom row). **D** Same as **C** but for bouts of optogenetic silencing with GtACR1. In all plots, times series curves and envelope correspond to population mean $\pm$ 95% confidence interval (500-sample bootstrap). Horizontal axis corresponds to non-dimensionalized wingbeat (wb) cycle time, with 0 and 1 corresponding to the dorsal-most points of the wingstroke. See Table S3 for full fly genotypes.

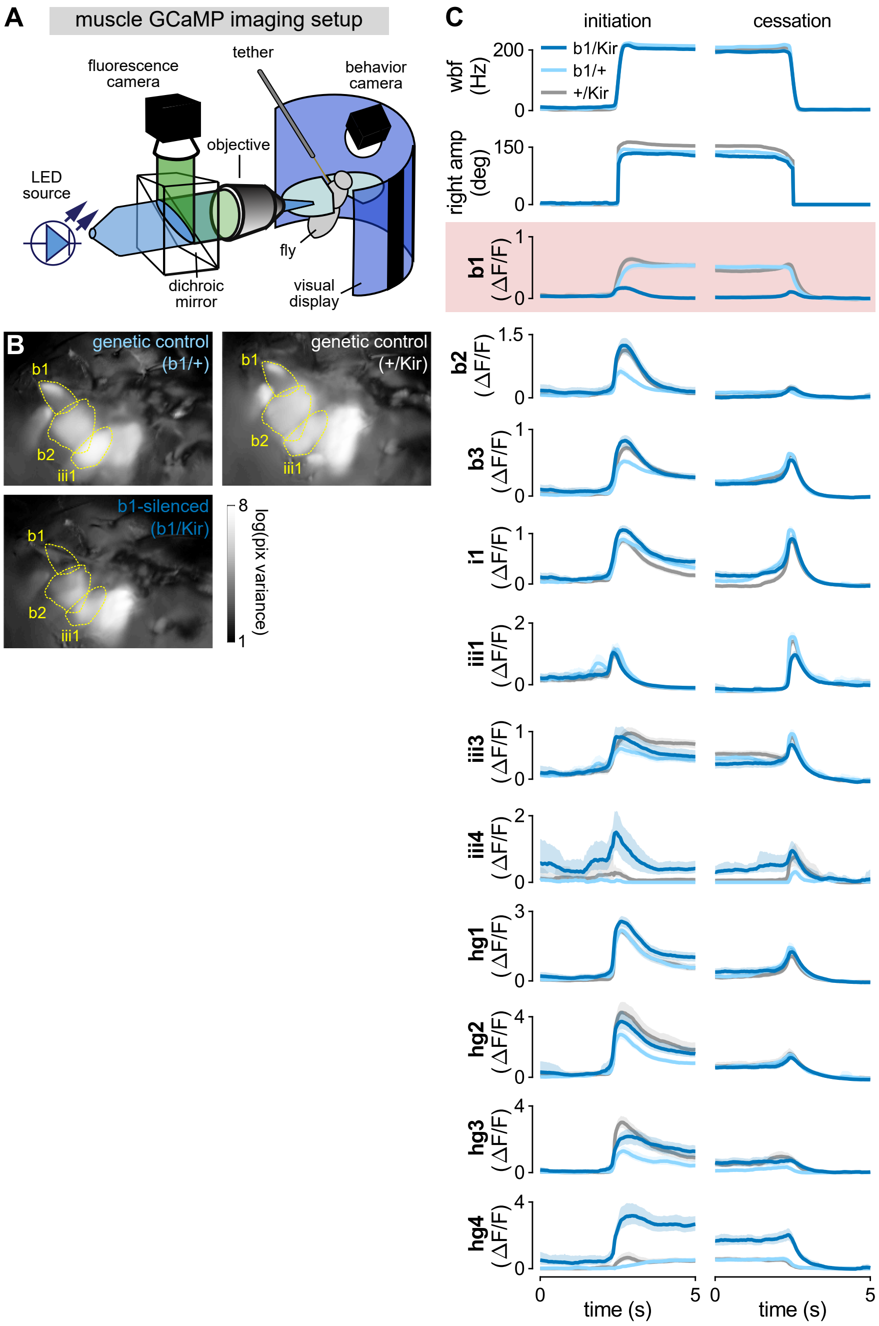

Figure S3: Calcium imaging of muscle activity. **A** Schematic for muscle imaging apparatus, from (*30*). The steering muscles on the right side of tethered flies are imaged using epifluorescent optics while the flies are presented with visual stimuli on the display screen. Simultaneously, the stroke amplitude and wingbeat frequency are also recorded. **B** Log-transformed pixel variance in steering muscle imaging across ∼200 seconds of continuous closed loop stripe fixation for genetic controls (top row) and b1-silenced flies (bottom). Yellow dashed lines show outlines of the b1, b2, and iii1 muscles as reference. **C** Mean wing kinematics and muscle activity at the initiation (left) and cessation (right) of flight for b1-silenced flies (dark blue; N=18 flies) and two genetic controls (light blue and gray; N=17 and N=20 flies). See Table S3 for full fly genotypes.

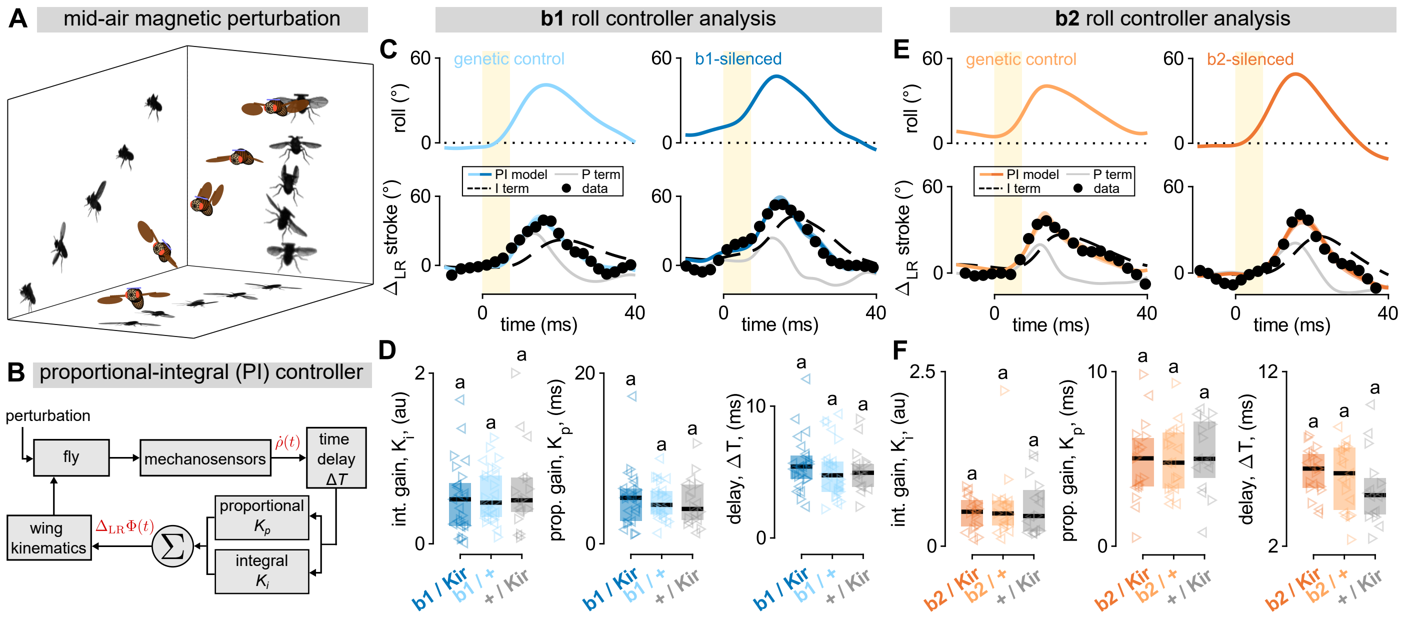

Figure S4: Chronic b1 and b2 inhibition do not alter roll stabilization behavior. **A** Illustration of a fly experiencing and correcting for a leftward roll perturbation. The 3D rendered fly represents measured kinematics, with attached ferromagnetic pin false-colored blue. Walls show photomontages from the three high-speed cameras filming the perturbation event. **B** Generalized proportional-integral (PI) controller model for rapid flight stabilization, as in Equation S1 and Figure 3B. **C** Example roll right perturbation analyses for a genetic control fly (left) and a b1-silenced fly (right). Top row shows change in body roll angle over time (blue traces); bottom row shows measured change in left-right stroke amplitude difference over time (black dots) as well as PI controller model fit to data (blue traces with 95% CI). Bottom row plots also show contributions of individual model terms: P term (proportional; thin gray line) and I term (integral; dashed black line). Yellow bar indicates 7 ms magnetic pulse. **D** Summary statistics for PI controller model parameters from Equation S1—integral gain (K_i; left), proportional gain (K_p; center), and time delay (ΔT, right)—for b1-silenced flies (dark blue; N=25) and two genetic controls (light blue and gray; N=26 and 18). Each data point represents a roll perturbation movie, with left- and right-pointing triangles representing left and right roll perturbations, respectively. Horizontal black lines and boxes show median and interquartile range for each genotype. **E** Same as in C but with a genetic control (left) and b2-silenced fly (right). **F** Same as **D** but with b2-silenced flies (dark orange; N=19) and two genetic controls (light orange and gray; N=19 and 16). Lower case letters above data in both **D** and **F** indicate significance categories, determined via Kruskal-Wallis test with Bonferroni method multiple comparison (α=0.05). See Table S3 for full fly genotypes.

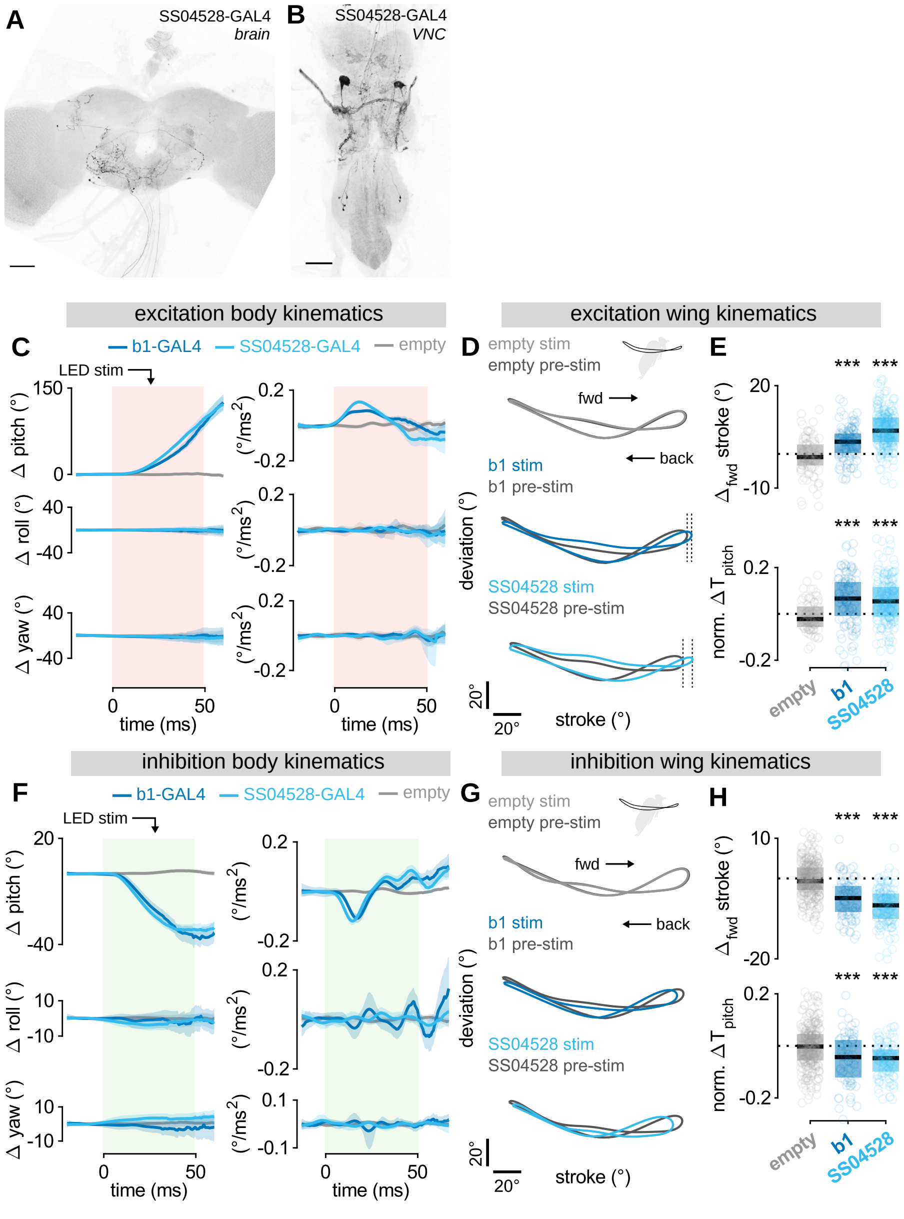

A
SS04528-GAL4
brain
B
SS04528-GAL4
VNC
excitation body kinematics
excitation wing kinematics
C
b1-GAL4
SS04528-GAL4
empty
LED stim
Δ pitch (°)
Δ roll (°)
Δ yaw (°)
(°/ms²)
time (ms)
D
empty stim
empty pre-stim
fwd
back
b1 stim
b1 pre-stim
SS04528 stim
SS04528 pre-stim
deviation (°)
stroke (°)
E
Δ_{fwd} stroke (°)
norm. ΔT_{pitch}
***
empty
b1
SS04528
inhibition body kinematics
inhibition wing kinematics
F
G
H

Figure S5: Two driver lines targeting the b1 motoneuron respond consistently to optogenetic manipulation. **A** Maximum intensity projection (MIP) image of the brain of a *UAS-CsChrimson* > *SS04528-GAL4* fly. Black corresponds to mVenus, light gray to DNCad (neuropil). Scale bar 50 µm. **B** MIP image of a *UAS-CsChrimson* > *SS04528-GAL4* fly VNC. Colors and scale bar as in **A**. **C** Body kinematics versus time in response to 50 ms optogenetic activation of *b1-GAL4* (dark blue; *N*=140 movies), *SS04528-GAL4* (light blue/cyan; *N*=272 movies), and *SS01062-GAL4* (aka *empty*; gray; *N*=108 movies) flies with *UAS-CsChrimson*, as in Figure 2A. Rows correspond to rotational degrees of freedom: pitch (top), roll (middle), and yaw (bottom). Columns give angular displacement (left) and angular acceleration (right). Data shown represent mean ± 95% confidence interval. **D** Wing kinematic data for movies in **C**. Plots show population-averaged wing tip angular position in the wingstrokes prior to (dark gray; "pre-stim") and during (light gray, dark blue, light blue/cyan; "stim") optogenetic activation. Vertical and horizontal scale bars provide 20° references for deviation and stroke angles, respectively. **E** Change in forward stroke angle (top) and normalized, wingbeat-averaged aerodynamic pitch torque (bottom) for wingbeats prior to and during optogenetic activation of *b1-GAL4* (dark blue), *SS04528-GAL4* (light blue/cyan), and *SS01062-GAL4* (gray) flies. Circles show raw data; box and horizontal line show interquartile range and median, respectively. Statistical significance determined via Wilcoxon signed-rank test (***, p<0.001; **, p<0.01; *, p<0.05). **F–H** Same as in **C–E** but with optogenetic silencing of *b1-GAL4* (dark blue; *N*=89 movies), *SS04528-GAL4* (light blue/cyan; *N*=146 movies), and *SS01062-GAL4* (gray; *N*=323 movies) flies. See Table S3 for full fly genotypes.

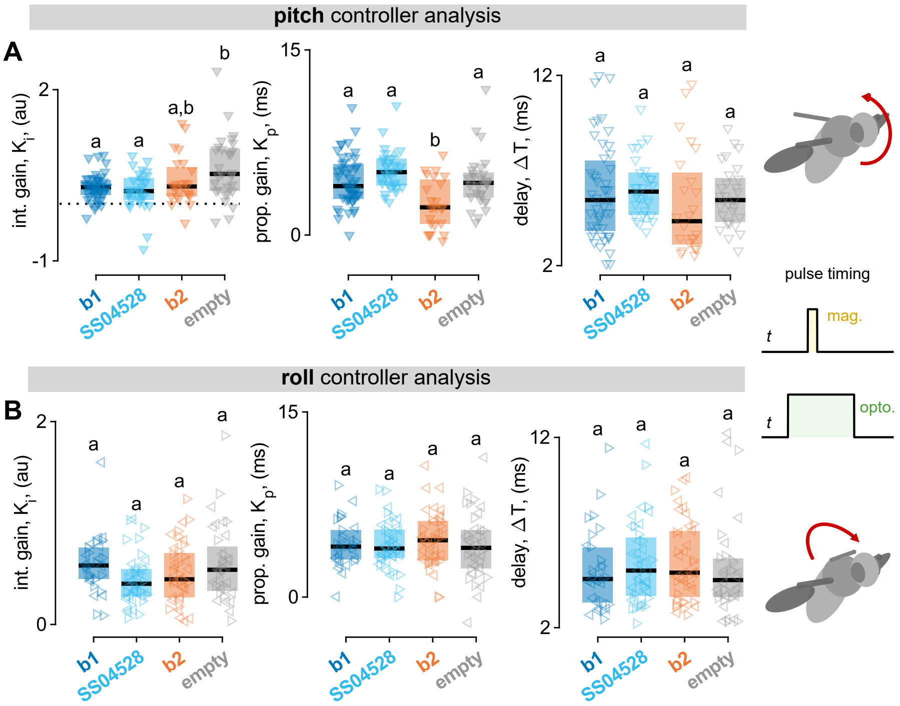

Figure S6: Combined optogenetic silencing and mechanical perturbation experiments. **A** PI controller coefficients—K_{i}(integral gain, left), K_{p}(proportional gain, middle), ΔT (time delay, right)—for fits to pitch perturbation movies wherein flies are exposed to a 50 ms LED pulse to induce optogenetic silencing followed by a 7 ms magnetic perturbation that begins 15 ms after the LED onset (pulse structure schematized on the right). Data shown for *UAS-GtACR1* crossed with *b1-GAL4* (dark blue; *N*=47 movies), *SS04528-GAL4* (alternate b1 MN driver line; light blue/cyan; *N*=30 movies), *b2-GAL4* (orange; *N*=24 movies), and *SS01062-GAL4* (aka *empty*; gray; *N*=29 movies) flies. Lower case letters above data indicate significance categories, determined via Kruskal-Wallis test with Bonferroni method multiple comparison (α=0.05). **B** Same as in **A** but for roll correction videos with the same structure of optogenetic pulse and magnetic perturbation. Data shown for *b1-GAL4* (dark blue; *N*=30 movies), *SS04528-GAL4* (alternate b1 MN driver line; light blue/cyan; *N*=40 movies), *b2-GAL4* (orange; *N*=40 movies), and *SS01062-GAL4* (aka *empty*; gray; *N*=36 movies) flies. See Table S3 for full fly genotypes.

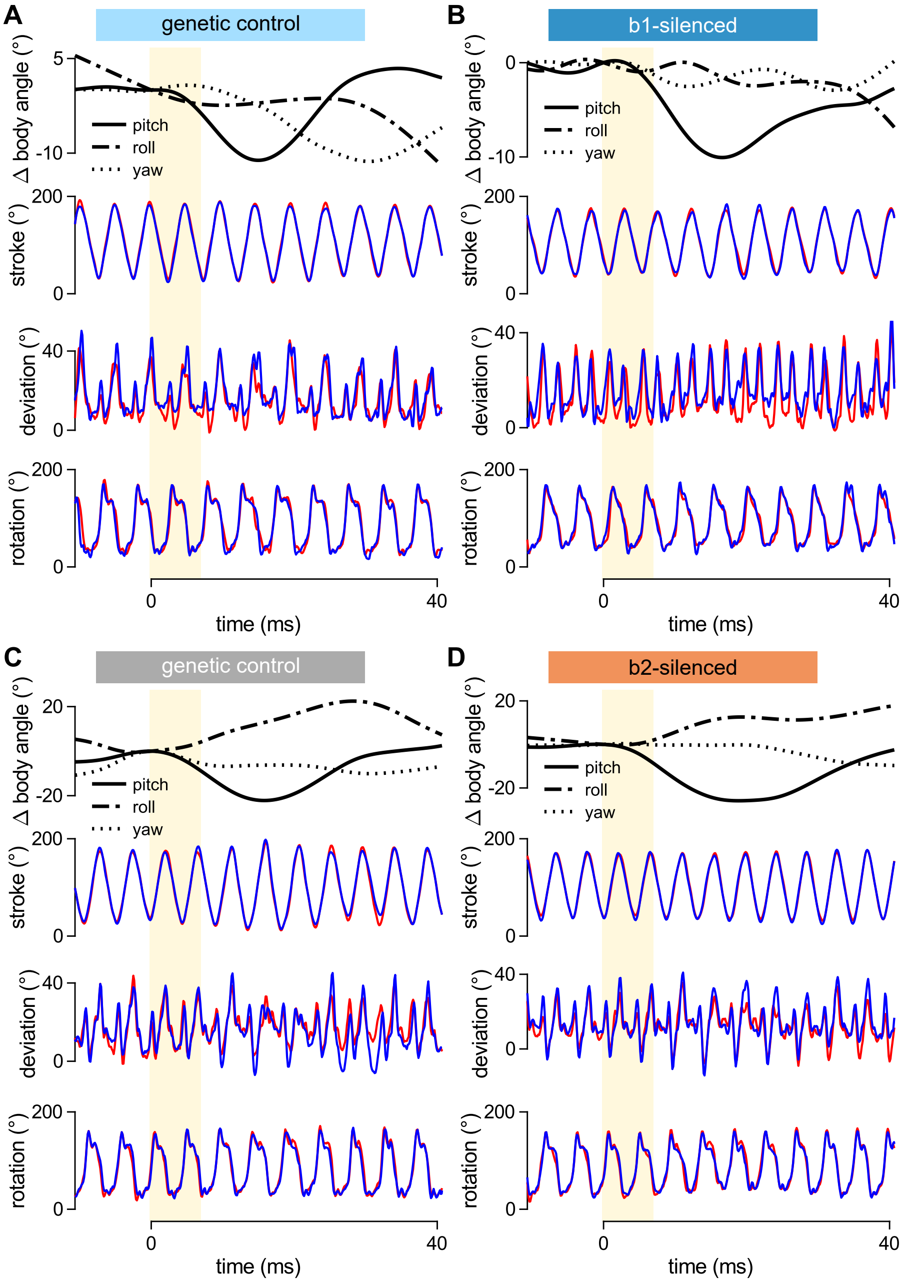

Figure S7: Full kinematics for example perturbations. Related to Figure 3. **A–D** Body and wing Euler angles as a function of time for example perturbation events shown in Figure 3C,E. In each panel, top row show the body Euler angles pitch (solid lines), roll (dashed-dotted lines), and yaw (dotted lines). Bottom three rows show wing Euler angles (stroke, top; deviation, middle; rotation, bottom), with red and blue lines denoting right and left wing, respectively. Yellow bar indicates period of 7 ms magnetic pulse. See Table S3 for full fly genotypes.

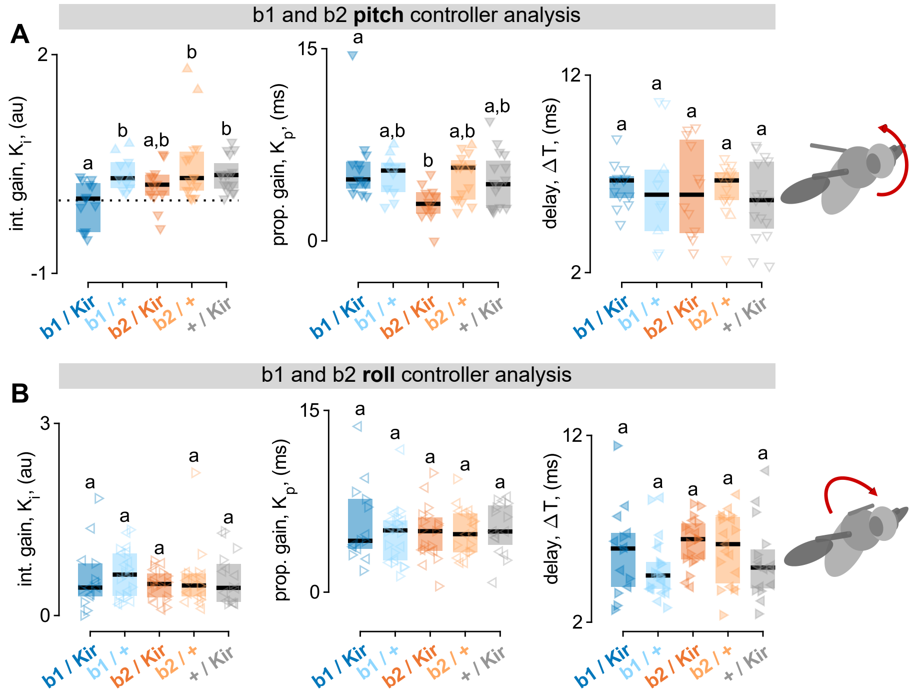

Figure S8: Perturbation experiments with *5XUAS-Kir2.1* effector line. **A** PI controller coefficients—K_{i}(integral gain, left), K_{p}(proportional gain, middle), ΔT(time delay, right)—for fits to pitch perturbation movies as in Figure 3D,F. Data shown for *b1/Kir2.1* (dark blue; *N*=18 movies), *b1/+* (light blue; *N*=16 movies), *b2/Kir2.1* (dark orange; *N*=17 movies), *b2/+* (light orange; *N*=22 movies), and *+/Kir2.1* (gray; *N*=22 movies). Data for b2 flies and genetic controls is the same as in Figure 3F. Lower case letters above data indicate significance categories, determined via Kruskal-Wallis test with Bonferroni method multiple comparison (α=0.05). **B** Same as in **A** but for roll correction videos. Data shown for *b1/Kir2.1* (dark blue; *N*=14 movies), *b1/+* (light blue; *N*=22 movies), *b2/Kir2.1* (dark orange; *N*=19 movies), *b2/+* (light orange; *N*=19 movies), and *+/Kir2.1* (gray; *N*=16 movies). Data for b2 flies and genetic controls is the same as in Figure S4F. See Table S3 for full fly genotypes.

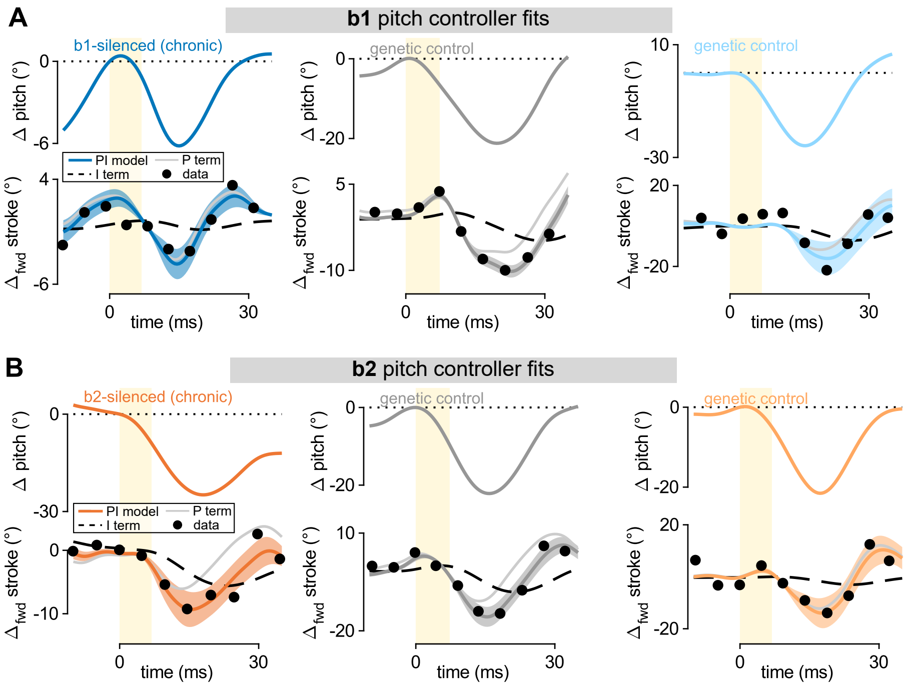

Figure S9: Additional example pitch perturbations and controller fits. Related to Figure 3. **A** Example pitch perturbation analyses for a b1-silenced fly (left) and two genetic control flies (middle and right), as in Figure 3C. Top row shows change in body pitch angle over time (blue and gray traces); bottom row shows measured change in forward stroke angle over time (black dots), PI controller model fit to data (thick blue and gray traces with 95% CI), P term (proportional; thin gray line), and I term (integral; dashed black line). Yellow bar indicates 7 ms magnetic pulse. Example perturbation events selected to match the genotype-level median for integral gain, K_i. **B** Same as **A** but for a b2-silenced fly (left) and two genetic control flies (middle and right), as in Figure 3E. Example perturbation events selected to match the genotype-level median for proportional gain, K_p. See Table S3 for full fly genotypes.

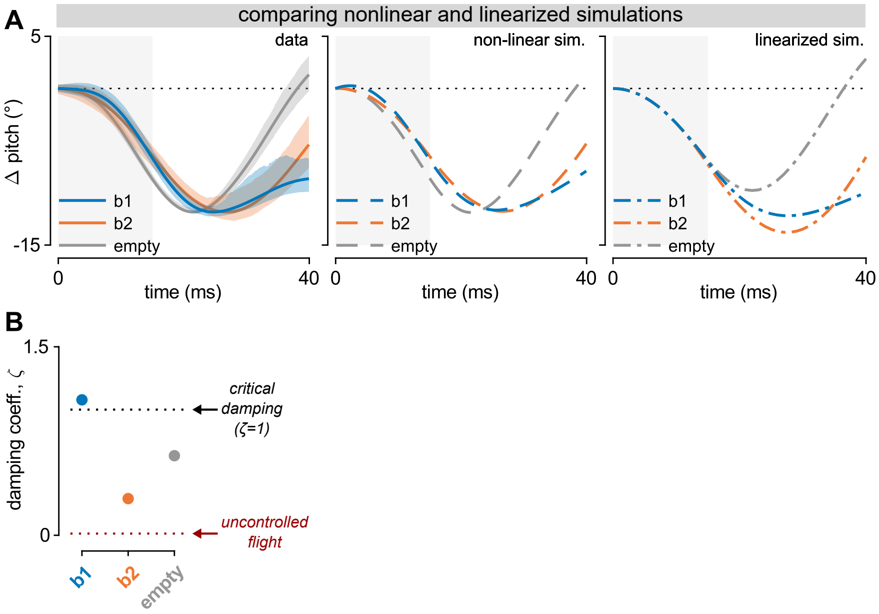

Figure S10: Linearized model for closed-loop dynamics. **A** Change in body pitch angle over time as measured from experimental data (population average with 95% CI envelope; left), flapping flight simulation (middle), and linearized dynamical model (right). Curve colors correspond to different genotypes: *b1-GAL4 > GtACR1* ("b1"; blue), *b2-GAL4 > GtACR1* ("b2"; orange), and *empty > GtACR1* ("empty"; gray). Gray bar represents the 15 ms simultaneous LED and magnetic field stimuli, which optogenetically silence and impose external torque, respectively. Left and middle plots are the same data as in Figure 3H. **B** Damping ratio, ζ, estimated from linearized model of closed-loop dynamics for b1-silenced (blue), b2-silenced (orange), and genetic control (gray) flies. See Table S3 for full fly genotypes.

Name	Genotype	Source
b1-GAL4 (*MB258C*)	*R71D08-p65ADZp* (attP2), *R33E02-ZpGAL4DBD* (VK00027)	gift from Troy Shirangi
b2-GAL4	*VT40232-p65ADZp* (attp40) ; *VT23830-ZpGAL4DBD* (attp2)	gift from Anne von Phillips-born (*36*)
empty (*SS01062*)	*R24A03-p65ADZp* (attP40) ; *R74C01-ZpGDBD* (attP2)	gift from Gwyneth Card (*40*)
SS04528	*R19G08-p65ADZp* (attP40) ; *R47F01-ZpGDBD* (attP2)	gift from Gwyneth Card (*40*)
UAS-CsChrimson	*20XUAS-CsChrimson-mVenus* (attP18)	gift from Gwyneth Card (*37*)
UAS-GtACR1	*pJFRC7-20XUAS-IVS-GtACR1-EYFP* (attP40)	gift from Nilay Yapici (*38*)
10XUAS-Kir2.1	*pJFRC49-10XUAS-IVS-eGFPKir2.1* (attP2)	gift from Troy Shirangi
5XUAS-Kir2.1	*pJFRC49-5XUAS-IVS-eGFPKir2.1* (attP2)	gift from David Stern
UAS-GFP	*10XUAS-IVS-mCD8::GFP* (attP40)	BDSC #32186
R39E01-LexA	*R39E01-LexA* (attP40)	BDSC #52776
LexAop-GCaMP6f	*13XLexAop2-IVS-GCaMP6f-p10* (su(Hw)attP5)	BDSC #44277
b1-GAL4 ∩ *tshLexA*	*tshLexA* (attP40) ; *R71D08-p65ADZp* (attP2), *R33E02-ZpGAL4DBD* (VK00027)	from Troy Shirangi
8XLexAop2-FlpL ∩ *10XUAS-FRT>STOP>FRT-Kir2.1*	*w+* ; *pJFRC79-8XLexAop2-FlpL* (attP40) ; *pJFRC56-10XUAS-FRT > STOP > FRT-kir2.1-gfp* (attP2)	from Troy Shirangi

Table S1: Fly stocks

Data shown in	Experiment type	Abbreviated genotype	Full genotype
Figure 1B	VNC anatomy	*b1-GAL4*	*20XUAS-CsChrimson-mVenus (attP18)/+ ; +/+ ; R71D08-p65ADZp (attP2) , R33E02-ZpGAL4DBD (VK00027)/+*
Figure 1C	VNC anatomy	*b2-GAL4*	*20XUAS-CsChrimson-mVenus (attP18)/+ ; VT40232-p65ADZp (attp40)/+ ; VT23830-ZpGAL4DBD (attp2)/+*
Figure 1G	opto. activation	*b1-GAL4 > CsChrimson*	*20XUAS-CsChrimson-mVenus (attP18)/+ ; +/+ ; R71D08-p65ADZp (attP2) , R33E02-ZpGAL4DBD (VK00027)/+*
Figure 1H	opto. silencing	*b1-GAL4 > GtACR1*	*w ; UAS-GtACR1 (attP40)/+ ; R71D08-p65ADZp (attP2) , R33E02-ZpGAL4DBD (VK00027)/+*
Figure 2A–C	opto. activation (control)	*empty*	*20XUAS-CsChrimson-mVenus (attP18)/+ ; R24A03-p65ADZp (attP40)/+ ; R74C01-ZpGdbd (attP2)/+*
Figure 2A–C	opto. activation	*b1*	*20XUAS-CsChrimson-mVenus (attP18)/+ ; +/+ ; R71D08-p65ADZp (attP2) , R33E02-ZpGAL4DBD (VK00027)/+*
Figure 2A–C	opto. activation	*b2*	*20XUAS-CsChrimson-mVenus (attP18)/+ ; VT40232-p65ADZp (attp40)/+ ; VT23830-ZpGAL4DBD (attp2)/+*
Figure 2D–F	opto. silencing (control)	*empty*	*w ; UAS-GtACR1 (attP40)/R24A03-p65ADZp (attP40) ; R74C01-ZpGdbd (attP2)/+*
Figure 2D–F	opto. silencing	*b1*	*w ; UAS-GtACR1 (attP40)/+ ; R71D08-p65ADZp (attP2) , R33E02-ZpGAL4DBD (VK00027)/+*
Figure 2D–F	opto. silencing	*b2*	*w ; UAS-GtACR1 (attP40)/VT40232-p65ADZp (attp40) ; VT23830-ZpGAL4DBD (attp2)/+*
Figure 3C,D	chronic silencing and pitch pert.	*b1-silenced* or *b1/Kir*	*w ; pJFRC79-8XLexAop2-FlpL (attP40)/+ ; pJFRC56-10XUAS-FRT > STOP > FRT-kir2.1-gfp (attP2)/R71D08-p65ADZp (attP2) , R33E02-ZpGAL4DBD (VK00027)*
Figure 3C,D	chronic silencing and pitch pert. (control)	*genetic control* or *b1/+*	*w ; +/+ ; R71D08-p65ADZp (attP2) , R33E02-ZpGAL4DBD (VK00027)/+*
Figure 3D	chronic silencing and pitch pert. (control)	*+/Kir*	*w ; pJFRC79-8XLexAop2-FlpL (attP40)/+ ; pJFRC56-10XUAS-FRT > STOP > FRT-kir2.1-gfp (attP2)/+*
Figure 3E,F	chronic silencing and pitch pert.	*b2-silenced* or *b2/Kir*	*w ; VT40232-p65ADZp (attp40)/+ ; pJFRC49-5XUAS-IVS-eGFPKir2.1 (attP2)/VT23830-ZpGAL4DBD (attp2)*
Figure 3E	chronic silencing and pitch pert. (control)	*b2/+*	*w ; VT40232-p65ADZp (attp40)/+ ; VT23830-ZpGAL4DBD (attp2)/+*
Figure 3E,F	chronic silencing and pitch pert. (control)	*genetic control* or *+/Kir*	*w ; +/+ ; pJFRC49-5XUAS-IVS-eGFPKir2.1 (attP2)/+*
Figure 4B,C	opto. silencing and pitch pert. (control)	*genetic control* or *empty*	*w ; UAS-GtACR1 (attP40)/R24A03-p65ADZp (attP40) ; R74C01-ZpGdbd (attP2)/+*
Figure 4B,C	opto. silencing and pitch pert.	*b1-silenced* or *b1*	*w ; UAS-GtACR1 (attP40)/+ ; R71D08-p65ADZp (attP2) , R33E02-ZpGAL4DBD (VK00027)/+*
Figure 4B,C	opto. silencing and pitch pert.	*b2-silenced* or *b2*	*w ; UAS-GtACR1 (attP40)/VT40232-p65ADZp (attp40) ; VT23830-ZpGAL4DBD (attp2)/+*

Table S2: Full genotype of flies used in experiments (main text figures).

**Data shown in**	**Experiment type**	**Abbreviated genotype**	**Full genotype**
Figure S1A,B	CNS anatomy	*b1-GAL4*	*20XUAS-CsChrimson-mVenus (attP18)/+ ; +/+ ; R71D08-p65ADZp (attP2) , R33E02-ZpGAL4DBD (VK00027)/+*
Figure S1C,D	CNS anatomy	*b2-GAL4*	*20XUAS-CsChrimson-mVenus (attP18)/+ ; VT40232-p65ADZp (attp40)/+ ; VT23830-ZpGAL4DBD (attp2)/+*
Figure S1E,F	phalloidin anatomy	*b1-GAL4*	*w ; 10XUAS-IVS-mCD8::GFP (attP40)/+ ; R71D08-p65ADZp (attP2) , R33E02-ZpGAL4DBD (VK00027)/+*
Figure S2A,C	opto. activation (control)	*empty*	*20XUAS-CsChrimson-mVenus (attP18)/+ ; R24A03-p65ADZp (attP40)/+ ; R74C01-ZpGdbd (attP2)/+*
Figure S2A,C	opto. activation	*b1*	*20XUAS-CsChrimson-mVenus (attP18)/+ ; +/+ ; R71D08-p65ADZp (attP2) , R33E02-ZpGAL4DBD (VK00027)/+*
Figure S2A,C	opto. activation	*b2*	*20XUAS-CsChrimson-mVenus (attP18)/+ ; VT40232-p65ADZp (attp40)/+ ; VT23830-ZpGAL4DBD (attp2)/+*
Figure S2A,C	opto. activation	*SS04528*	*20XUAS-CsChrimson-mVenus (attP18)/+ ; R19G08-p65ADZp (attP40)/+ ; R47F01-ZpGDBD (attP2)/+*
Figure S2B,D	opto. silencing (control)	*empty*	*w ; UAS-GtACR1 (attP40)/R24A03-p65ADZp (attP40) ; R74C01-ZpGdbd (attP2)/+*
Figure S2B,D	opto. silencing	*b1*	*w ; UAS-GtACR1 (attP40)/+ ; R71D08-p65ADZp (attP2) , R33E02-ZpGAL4DBD (VK00027)/+*
Figure S2B,D	opto. silencing	*b2*	*w ; UAS-GtACR1 (attP40)/VT40232-p65ADZp (attp40) ; VT23830-ZpGAL4DBD (attp2)/+*
Figure S2B,D	opto. silencing	*SS04528*	*w ; UAS-GtACR1 (attP40)/R19G08-p65ADZp (attP40) ; R47F01-ZpGDBD (attP2)/+*
Figure S3	muscle imaging	*b1-silenced* or *b1/Kir*	*w ; 13XLexAop2-IVS-GCaMP6f-p10 (su(Hw)attP5)/R39E01-LexA (attP40) ; pJFRC49-10XUAS-IVS-eGFPKir2.1 (attP2)/R71D08-p65ADZp (attP2) , R33E02-ZpGAL4DBD (VK00027)*
Figure S3	muscle imaging	*genetic control* or *b1/+*	*w ; 13XLexAop2-IVS-GCaMP6f-p10 (su(Hw)attP5)/R39E01-LexA (attP40) ; R71D08-p65ADZp (attP2) , R33E02-ZpGAL4DBD (VK00027)/+*
Figure S3	muscle imaging	*genetic control* or *+/Kir*	*w ; 13XLexAop2-IVS-GCaMP6f-p10 (su(Hw)attP5)/R39E01-LexA (attP40) ; pJFRC49-10XUAS-IVS-eGFPKir2.1 (attP2)/+*

Data shown in	Experiment type	Abbreviated genotype	Full genotype
Figure S4C,D	chronic silencing and roll pert.	*b1-silenced* or *b1/Kir*	*w ; pJFRC79-8XLexAop2-FlpL (attP40)/+ ; pJFRC56-10XUAS-FRT > STOP > FRT-kir2.1-gfp (attP2)/R71D08-p65ADZp (attP2) , R33E02-ZpGAL4DBD (VK00027)*
Figure S4C,D	chronic silencing and roll pert. (control)	*genetic control* or *b1/+*	*w ; +/+ ; R71D08-p65ADZp (attP2) , R33E02-ZpGAL4DBD (VK00027)/+*
Figure S4D	chronic silencing and roll pert. (control)	*+/Kir*	*w ; pJFRC79-8XLexAop2-FlpL (attP40)/+ ; pJFRC56-10XUAS-FRT > STOP > FRT-kir2.1-gfp (attP2)/+*
Figure S4E,F	chronic silencing and roll pert.	*b2-silenced* or *b2/Kir*	*w ; VT40232-p65ADZp (attp40)/+ ; pJFRC49-5XUAS-IVS-eGFPKir2.1 (attP2)/VT23830-ZpGAL4DBD (attp2)*
Figure S4E,F	chronic silencing and roll pert. (control)	*genetic control* or *b2/+*	*w ; VT40232-p65ADZp (attp40)/+ ; VT23830-ZpGAL4DBD (attp2)/+*
Figure S4F	chronic silencing and roll pert. (control)	*+/Kir*	*w ; +/+ ; pJFRC49-5XUAS-IVS-eGFPKir2.1 (attP2)/+*
Figure S5A,B	anatomy	*SS04528*	*20XUAS-CsChrimson-mVenus (attP18)/+ ; R19G08-p65ADZp (attP40)/+ ; R47F01-ZpGDBD (attP2)/+*
Figure S5C–E	opto. activation (control)	*empty*	*20XUAS-CsChrimson-mVenus (attP18)/+ ; R24A03-p65ADZp (attP40)/+ ; R74C01-ZpGdbd (attP2)/+*
Figure S5C–E	opto. activation	*b1*	*20XUAS-CsChrimson-mVenus (attP18)/+ ; +/+ ; R71D08-p65ADZp (attP2) , R33E02-ZpGAL4DBD (VK00027)/+*
Figure S5C–E	opto. activation	*SS04528*	*20XUAS-CsChrimson-mVenus (attP18)/+ ; R19G08-p65ADZp (attP40)/+ ; R47F01-ZpGDBD (attP2)/+*
Figure S5F–H	opto. silencing (control)	*empty*	*w ; UAS-GtACR1 (attP40)/R24A03-p65ADZp (attP40) ; R74C01-ZpGdbd (attP2)/+*
Figure S5F–H	opto. silencing	*b1*	*w ; UAS-GtACR1 (attP40)/+ ; R71D08-p65ADZp (attP2) , R33E02-ZpGAL4DBD (VK00027)/+*
Figure S5F–H	opto. silencing	*SS04528*	*w ; UAS-GtACR1 (attP40)/R19G08-p65ADZp (attP40) ; R47F01-ZpGDBD (attP2)/+*
Figure S6A	opto. silencing and pitch pert.	*b1*	*w ; UAS-GtACR1 (attP40)/+ ; R71D08-p65ADZp (attP2) , R33E02-ZpGAL4DBD (VK00027)/+*
Figure S6A	opto. silencing and pitch pert.	*SS04528*	*w ; UAS-GtACR1 (attP40)/R19G08-p65ADZp (attP40) ; R47F01-ZpGDBD (attP2)/+*
Figure S6A	opto. silencing and pitch pert.	*b2*	*w ; UAS-GtACR1 (attP40)/VT40232-p65ADZp (attp40) ; VT23830-ZpGAL4DBD (attp2)/+*
Figure S6A	opto. silencing and pitch pert. (control)	*empty*	*w ; UAS-GtACR1 (attP40)/R24A03-p65ADZp (attP40) ; R74C01-ZpGdbd (attP2)/+*

**Data shown in**	**Experiment type**	**Abbreviated genotype**	**Full genotype**
Figure S6B	opto. silencing and roll pert.	*b1*	*w ; UAS-GtACR1 (attP40)/+ ; R71D08-p65ADZp (attP2) , R33E02-ZpGAL4DBD (VK00027)/+*
Figure S6B	opto. silencing and roll pert.	*SS04528*	*w ; UAS-GtACR1 (attP40)/R19G08-p65ADZp (attP40) ; R47F01-ZpGDBD (attP2)/+*
Figure S6B	opto. silencing and roll pert.	*b2*	*w ; UAS-GtACR1 (attP40)/VT40232-p65ADZp (attp40) ; VT23830-ZpGAL4DBD (attp2)/+*
Figure S6B	opto. silencing and roll pert. (control)	*empty*	*w ; UAS-GtACR1 (attP40)/R24A03-p65ADZp (attP40) ; R74C01-ZpGdbd (attP2)/+*
Figure S7A	chronic silencing and pitch pert. (control)	*genetic control*	*w ; +/+ ; R71D08-p65ADZp (attP2) , R33E02-ZpGAL4DBD (VK00027)/+*
Figure S7B	chronic silencing and pitch pert.	*b1-silenced*	*w ; pJFRC79-8XLexAop2-FlpL (attP40)/+ ; pJFRC56-10XUAS-FRT > STOP > FRT-kir2.1-gfp (attP2)/R71D08-p65ADZp (attP2) , R33E02-ZpGAL4DBD (VK00027)*
Figure S7C	chronic silencing and pitch pert. (control)	*genetic control*	*w ; +/+ ; pJFRC49-5XUAS-IVS-eGFPKir2.1 (attP2)/+*
Figure S7D	chronic silencing and pitch pert.	*b2-silenced*	*w ; VT40232-p65ADZp (attp40)/+ ; pJFRC49-5XUAS-IVS-eGFPKir2.1 (attP2)/VT23830-ZpGAL4DBD (attp2)*
Figure S8A	chronic silencing and pitch pert.	*b1/Kir*	*w ; +/+ ; pJFRC49-5XUAS-IVS-eGFPKir2.1 (attP2)/R71D08-p65ADZp (attP2) , R33E02-ZpGAL4DBD (VK00027)*
Figure S8A	chronic silencing and pitch pert. (control)	*b1/+*	*w ; +/+ ; R71D08-p65ADZp (attP2) , R33E02-ZpGAL4DBD (VK00027)/+*
Figure S8A	chronic silencing and pitch pert.	*b2/Kir*	*w ; VT40232-p65ADZp (attp40)/+ ; pJFRC49-5XUAS-IVS-eGFPKir2.1 (attP2)/VT23830-ZpGAL4DBD (attp2)*
Figure S8A	chronic silencing and pitch pert. (control)	*b2/+*	*w ; VT40232-p65ADZp (attp40)/+ ; VT23830-ZpGAL4DBD (attp2)/+*
Figure S8A	chronic silencing and pitch pert. (control)	*+/Kir*	*w ; +/+ ; pJFRC49-5XUAS-IVS-eGFPKir2.1 (attP2)/+*

Data shown in	Experiment type	Abbreviated genotype	Full genotype
Figure S8B	chronic silencing and roll pert.	*b1/Kir*	*w ; +/+ ; pJFRC49-5XUAS-IVS-eGFPKir2.1 (attP2)/R71D08-p65ADZp (attP2) , R33E02-ZpGAL4DBD (VK00027)*
Figure S8B	chronic silencing and roll pert. (control)	*b1/+*	*w ; +/+ ; R71D08-p65ADZp (attP2) , R33E02-ZpGAL4DBD (VK00027)/+*
Figure S8B	chronic silencing and roll pert.	*b2/Kir*	*w ; VT40232-p65ADZp (attp40)/+ ; pJFRC49-5XUAS-IVS-eGFPKir2.1 (attP2)/VT23830-ZpGAL4DBD (attp2)*
Figure S8B	chronic silencing and roll pert. (control)	*b2/+*	*w ; VT40232-p65ADZp (attp40)/+ ; VT23830-ZpGAL4DBD (attp2)/+*
Figure S8B	chronic silencing and roll pert. (control)	*+/Kir*	*w ; +/+ ; pJFRC49-5XUAS-IVS-eGFPKir2.1 (attP2)/+*
Figure S9A	chronic silencing and pitch pert.	*b1-silenced*	*w ; pJFRC79-8XLexAop2-FlpL (attP40)/+ ; pJFRC56-10XUAS-FRT > STOP > FRT-kir2.1-gfp (attP2)/R71D08-p65ADZp (attP2) , R33E02-ZpGAL4DBD (VK00027)*
Figure S9A	chronic silencing and pitch pert. (control)	*genetic control (gray)*	*w ; pJFRC79-8XLexAop2-FlpL (attP40)/+ ; pJFRC56-10XUAS-FRT > STOP > FRT-kir2.1-gfp (attP2)/+*
Figure S9A	chronic silencing and pitch pert. (control)	*genetic control (light blue)*	*w ; +/+ ; R71D08-p65ADZp (attP2) , R33E02-ZpGAL4DBD (VK00027)/+*
Figure S9B	chronic silencing and pitch pert.	*b2-silenced*	*w ; VT40232-p65ADZp (attp40)/+ ; pJFRC49-5XUAS-IVS-eGFPKir2.1 (attP2)/VT23830-ZpGAL4DBD (attp2)*
Figure S9B	chronic silencing and pitch pert. (control)	*genetic control (gray)*	*w ; +/+ ; pJFRC49-5XUAS-IVS-eGFPKir2.1 (attP2)/+*
Figure S9B	chronic silencing and pitch pert. (control)	*genetic control (light orange)*	*w ; VT40232-p65ADZp (attp40)/+ ; VT23830-ZpGAL4DBD (attp2)/+*
Figure S10A,B	opto. silencing and pitch pert. (control)	*empty*	*w ; UAS-GtACR1 (attP40)/R24A03-p65ADZp (attP40) ; R74C01-ZpGdbd (attP2)/+*
Figure S10A,B	opto. silencing and pitch pert.	*b1*	*w ; UAS-GtACR1 (attP40)/+ ; R71D08-p65ADZp (attP2) , R33E02-ZpGAL4DBD (VK00027)/+*
Figure S10A,B	opto. silencing and pitch pert.	*b2*	*w ; UAS-GtACR1 (attP40)/VT40232-p65ADZp (attp40) ; VT23830-ZpGAL4DBD (attp2)/+*

Table S3: Full genotype of flies used in experiments (SI figures).

Symbol	Definition	Value
m	body mass	1.34 mg (*61*)
I_{pitch}	pitch moment of inertia	0.506 mg mm^2 (*68*)
R	wing span length	2.13 mm
$\overline{c}$	mean wing chord length	0.7 mm
S	wing area	5.74 mm^2 (*61*)
$\hat{r}_2^2(S)$	non-dimensionalized second moment of wing area	0.313 (*68*)
f	wingbeat frequency	225 Hz
ϕ_0	stroke angle offset	97.3°
ϕ_m	stroke angle amplitude	67.6°
K	stroke angle waveform parameter	0.7 (*3*)
θ_0	deviation angle offset	0°
θ_m	deviation angle amplitude	0°
δ_θ	deviation angle phase shift	0°
η_0	rotation angle offset	90.0°
η_m	rotation angle amplitude	67.3°
δ_η	rotation angle phase shift	–85.0°
C	rotation angle waveform parameter	2.4 (*3*)
ρ	air density	1.2 kg m^{-3}
g	gravitational acceleration	9.8 m s^{-2}
C_{friction}	body pitch rotational drag	0.52 mg mm^2 (*68*)
C_{rot}	rotational force coefficient	1.57 (*58*)
$C_{\text{L}_{\text{max}}}$	maximum lift coefficient	1.8 (*60, 63*)
$C_{\text{D}_{\text{max}}}$	maximum drag coefficient	3.4 (*60, 63*)
C_{D_0}	minimum drag coefficient	0.4 (*60, 63*)
T_{pulse}	magnetic perturbation duration	7 ms or 15 ms
θ_{pin}	angle between body and pin	45°
r_{hinge}	distance from body cm to wing hinge	0.22 mm

Table S4: Model parameters.

Movie S1. Optogenetic activation of a b1-GAL4 > CsChrimson fly. High speed video footage of a *b1-GAL4 > CsChrimson* fly undergoing a 50 ms bout of red light stimulus (indicated by red square in corner of screen). The three panels show two side views (left and right) and one top view (middle). Panel in bottom left corner indicates time in the recording relative to the onset of light stimulus (t=0 ms), measured in milliseconds. This video corresponds to the photomontage labeled “1” in Figure 1G.

Movie S2. Optogenetic activation of a b1-GAL4 > CsChrimson fly (second example). A second example of a *b1-GAL4 > CsChrimson* fly undergoing a optogenetic stimulation, corresponding to the photomontage labeled “2” in Figure 1G.

Movie S3. Optogenetic silencing of a b1-GAL4 > GtACR1 fly. High speed video footage of a *b1-GAL4 > GtACR1* fly undergoing a 50 ms bout of green light stimulus (indicated by green square in corner of screen). This video corresponds to the photomontage labeled “1” in Figure 1H.

Movie S4. Optogenetic silencing of a b1-GAL4 > GtACR1 fly (second example).A second example of a *b1-GAL4 > GtACR1* fly undergoing undergoing optogenetic inhibition, correspondng to the photomontage labeled “2” in Figure 1H.

Movie S5. Optogenetic activation of 24 b1-GAL4 > CsChrimson flies. Side views from 24 movies showing *b1-GAL4 > CsChrimson* flies undergoing a 50 ms bout of red light stimulus (indicated by red square in corner of screen).

Movie S6. Optogenetic activation of 24 b2-GAL4 > CsChrimson flies. Side views from 24 movies showing *b2-GAL4 > CsChrimson* flies undergoing a 50 ms bout of red light stimulus (indicated by red square in corner of screen).

Movie S7. Optogenetic activation of 24 SS01062-GAL4 > CsChrimson flies. Side views from 24 movies showing *SS01062-GAL4 > CsChrimson* flies (genetic control) undergoing a 50 ms bout of red light stimulus (indicated by red square in corner of screen).

Movie S8. Optogenetic inhibition of 24 b1-GAL4 > GtACR1 > flies. Side views from 24 movies showing *b1-GAL4 > GtACR1* flies undergoing a 50 ms bout of green light stimulus (indicated by green square in corner of screen).

Movie S9. Optogenetic inhibition of 24 b2-GAL4 > GtACR1 flies. Side views from 24 movies showing *b2-GAL4 > GtACR1* undergoing a 50 ms bout of green light stimulus (indicated by green square in corner of screen).

Movie S10. Optogenetic inhibition of 24 SS01062-GAL4 > GtACR1 flies. Side views from 24 movies showing *SS01062-GAL4 > GtACR1* flies (genetic control) undergoing a 50 ms bout of green light stimulus (indicated by green square in corner of screen).

Movie S11. Video data for pitch perturbation of genetic control group fly in Figure 3C. Video footage showing two side views (left and right) and one overhead view (middle) of a genetic control fly undergoing

a 7 ms magnetic pitch perturbation (indicated by yellow square the corner of each view). Bottom left corner shows time, measured in milliseconds, relative to the onset of the magnetic pulse at t=0 ms.

Movie S12. Video data for pitch perturbation of b1 motoneuron silenced fly in Figure 3C. Video footage showing two side views (left and right) and one overhead view (middle) of a b1-silenced fly undergoing a 7 ms magnetic pitch perturbation, as in Movie S11.

Movie S13. Video data for pitch perturbation of genetic control group fly in Figure 3E. Video footage showing two side views (left and right) and one overhead view (middle) of a genetic control fly undergoing a 7 ms magnetic pitch perturbation, as in Movies S11 and S12.

Movie S14. Video data for pitch perturbation of b2 motoneuron silenced fly in Figure 3C. Video footage showing two side views (left and right) and one overhead view (middle) of a b2-silenced fly undergoing a 7 ms magnetic pitch perturbation, as in Movies S11 and S12.

REFERENCES AND NOTES

1. M. H. Dickinson, C. T. Farley, R. J. Full, M. A. Koehl, R. Kram, S. Lehman, How animals move: An integrative view. *Science* **288**, 100–106 (2000).

2. M. S. Madhav, N. J. Cowan, The synergy between neuroscience and control theory: The nervous system as inspiration for hard control challenges. *Annu. Rev. Control Robot. Auton. Syst.* **3**, 243–267 (2020).

3. S. Chang, Z. J. Wang, Predicting fruit fly's sensing rate with insect flight simulations. *Proc. Natl. Acad. Sci. U.S.A.* **111**, 11246–11251 (2014).

4. M. Sun, Insect flight dynamics: Stability and control. *Rev. Mod. Phys.* **86**, 615–646 (2014).

5. H. Taha, M. Kiani, T. L. Hedrick, J. S. M. Greeter, Vibrational control: A hidden stabilization mechanism in insect flight. *Sci. Robot.* **5**, 1–12 (2020).

6. A. Fayyazuddin, M. H. Dickinson, Haltere afferents provide direct, electrotonic input to a steering motor neuron in the blowfly, Calliphora. *J. Neurosci.* **16**, 5225–5232 (1996).

7. F. O. Lehmann, J. Bartussek, Neural control and precision of flight muscle activation in Drosophila. *J. Comp. Physiol. A Neuroethol. Sens. Neural Behav. Physiol.* **203**, 1–14 (2017).

8. J. Putney, R. Conn, S. Sponberg, Precise timing is ubiquitous, consistent, and coordinated across a comprehensive, spike-resolved flight motor program. *Proc. Natl. Acad. Sci. U.S.A.* **116**, 26951–26960 (2019).

9. B. H. Dickerson, Timing precision in fly flight control: Integrating mechanosensory input with muscle physiology. *Proc. Biol. Sci.* **287**, 20201774 (2020).

10. L. Ristroph, A. J. Bergou, G. Ristroph, K. Coumes, G. J. Berman, J. Guckenheimer, Z. J. Wang, I. Cohen, Discovering the flight autostabilizer of fruit flies by inducing aerial stumbles. *Proc. Natl. Acad. Sci. U.S.A.* **107**, 4820–4824 (2010).

11. L. Ristroph, G. Ristroph, S. Morozova, A. J. Bergou, S. Chang, J. Guckenheimer, Z. J. Wang, I. Cohen, Active and passive stabilization of body pitch in insect flight. *J. R. Soc. Interface* **10**, 20130237 (2013).

12. S. C. Whitehead, T. Beatus, L. Canale, I. Cohen, Pitch perfect: How fruit flies control their body pitch angle. *J. Exp. Biol.* **218**, 3508–3519 (2015).

13. T. Beatus, J. M. Guckenheimer, I. Cohen, Controlling roll perturbations in fruit flies. *J. R. Soc. Interface* **12**, 20150075 (2015).

14. D. B. Lockhart, L. H. Ting, Optimal sensorimotor transformations for balance. *Nat. Neurosci.* **10**, 1329–1336 (2007).

15. J. Lee, S. N. Sponberg, O. Y. Loh, A. G. Lamperski, R. J. Full, N. J. Cowan, Templates and anchors for antenna-based wall following in cockroaches and robots. *IEEE Trans. Robot.* **24**, 130–143 (2008).

16. E. Roth, M. B. Reiser, M. H. Dickinson, N. J. Cowan, A task-level model for optomotor yaw regulation in *Drosophila melanogaster*: A frequency-domain system identification approach, in *Proceedings of the IEEE Conference on Decision and Control* (IEEE, 2012), pp. 3721–3726.

17. S. Sefati, I. D. Neveln, E. Roth, T. R. T. Mitchell, J. B. Snyder, M. A. MacIver, E. S. Fortune, N. J. Cowan, Mutually opposing forces during locomotion can eliminate the tradeoff between maneuverability and stability. *Proc. Natl. Acad. Sci. U.S.A.* **110**, 18798–18803 (2013).

18. J. P. Dyhr, K. A. Morgansen, T. L. Daniel, N. J. Cowan, Flexible strategies for flight control: An active role for the abdomen. *J. Exp. Biol.* **216**, 1523–1536 (2013).

19. B. Schnell, P. T. Weir, E. Roth, A. L. Fairhall, M. H. Dickinson, Cellular mechanisms for integral feedback in visually guided behavior. *Proc. Natl. Acad. Sci. U.S.A.* **111**, 5700–5705 (2014).

20. S. B. Fuller, A. D. Straw, M. Y. Peek, R. M. Murray, M. H. Dickinson, Flying Drosophila stabilize their vision-based velocity controller by sensing wind with their antennae. *Proc. Natl. Acad. Sci. U.S.A.* **111**, E1182–E1191 (2014).

21. S. Sponberg, J. P. Dyhr, R. W. Hall, T. L. Daniel, Luminance-dependent visual processing enables moth flight in low light. *Science* **348**, 1245–1248 (2015).

22. E. E. Sutton, A. Demir, S. A. Stamper, E. S. Fortune, N. J. Cowan, Dynamic modulation of visual and electrosensory gains for locomotor control. *J. R. Soc. Interface* **13**, 20160057 (2016).

23. A. L. Stockl, K. Kihlstrom, S. Chandler, S. Sponberg, Comparative system identification of flower tracking performance in three hawkmoth species reveals adaptations for dim light vision. *Philos. Trans. R. Soc. Lond. B Biol. Sci.* **372**, 20160078 (2017).

24. R. J. Peterka, Sensory integration for human balance control. *Handb. Clin. Neurol.* **159**, 27–42 (2018).

25. I. Uyanik, S. Sefati, S. A. Stamper, K. A. Cho, M. M. Ankarali, E. S. Fortune, N. J. Cowan, Variability in locomotor dynamics reveals the critical role of feedback in task control. *eLife* **9**, e51219 (2020).

26. B. D. Pfeiffer, T.-T. B. Ngo, K. L. Hibbard, C. Murphy, A. Jenett, J. W. Truman, G. M. Rubin, Refinement of tools for targeted gene expression in Drosophila. *Genetics* **186**, 735–755 (2010).

27. J. H. Simpson, L. L. Looger, Functional imaging and optogenetics in Drosophila. *Genetics* **208**, 1291–1309 (2018).

28. J. R. Trimarchi, A. M. Schneiderman, The motor neurons innervating the direct flight muscles of Drosophila melanogaster are morphologically specialized. *J. Comp. Neurol.* **340**, 427–443 (1994).

29. M. H. Dickinson, M. S. Tu, The function of dipteran flight muscle. *Comp. Biochem. Physiol. A Physiol.* **116**, 223–238 (1997).

30. T. Lindsay, A. Sustar, M. Dickinson, The function and organization of the motor system controlling flight maneuvers in flies. *Curr. Biol.* **27**, 345–358 (2017).

31. G. Heide, K. G. Götz, Optomotor control of course and altitude in Drosophila melanogaster is correlated with distinct activities of at least three pairs of flight steering muscles. *J. Exp. Biol.* **199**, 1711–1726 (1996).

32. M. Tu, M. Dickinson, The control of wing kinematics by two steering muscles of the blowfly (Calliphora vicina). *J. Comp. Physiol. A* **178**, 813–830 (1996).

33. F. O. Lehmann, K. G. Götz, Activation phase ensures kinematic efficacy in flight-steering muscles of Drosophila melanogaster. *J. Comp. Physiol. A* **179**, 311–322 (1996).

34. C. N. Balint, M. H. Dickinson, The correlation between wing kinematics and steering muscle activity in the blowfly Calliphora vicina. *J. Exp. Biol.* **204**, 4213–4226 (2001).

35. A. Fayyazuddin, M. H. Dickinson, Convergent mechanosensory input structures the firing phase of a steering motor neuron in the blowfly, Calliphora. *J. Neurophysiol.* **82**, 1916–1926 (1999).

36. A. O'Sullivan, T. Lindsay, A. Prudnikova, B. Erdi, M. Dickinson, A. C. von Philipsborn, Multifunctional wing motor control of song and flight. *Curr. Biol.* **28**, 2705–2717.e4 (2018).

37. N. C. Klapoetke, Y. Murata, S. S. Kim, S. R. Pulver, A. Birdsey-Benson, Y. K. Cho, T. K. Morimoto, A. S. Chuong, E. J. Carpenter, Z. Tian, J. Wang, Y. Xie, Z. Yan, Y. Zhang, B. Y. Chow, B. Surek, M. Melkonian, V. Jayaraman, M. Constantine-Paton, G. K.-S. Wong, E. S. Boyden, Independent optical excitation of distinct neural populations. *Nat. Methods* **11**, 338–46 (2014).

38. F. Mohammad, J. C. Stewart, S. Ott, K. Chlebikova, J. Y. Chua, T. W. Koh, J. Ho, A. Claridge-Chang, Optogenetic inhibition of behavior with anion channelrhodopsins. *Nat. Methods* **14**, 271–274 (2017).

39. M. H. Dickinson, F.-O. O. Lehmann, S. P. Sane, Wing rotation and the aerodynamic basis of insect flight. *Science* **284**, 1954–1960 (1999).

40. S. Namiki, M. H. Dickinson, A. M. Wong, W. Korff, G. M. Card, The functional organization of descending sensory-motor pathways in drosophila. *eLife* **7**, e34272 (2018).

41. F. T. Muijres, M. J. Elzinga, J. M. Melis, M. H. Dickinson, Flies evade looming targets by executing rapid visually directed banked turns. *Science* **344**, 172–177 (2014).

42. R. A. Baines, J. P. Uhler, A. Thompson, S. T. Sweeney, M. Bate, Altered electrical properties in Drosophila neurons developing without synaptic transmission. *J. Neurosci.* **21**, 1523–1531 (2001).

43. T. R. Shirangi, D. L. Stern, J. W. Truman, Motor control of Drosophila courtship song. *Cell Rep.* **5**, 678–686 (2013).

44. L. Ristroph, G. J. Berman, A. J. Bergou, Z. J. Wang, I. Cohen, Automated hull reconstruction motion tracking (HRMT) applied to sideways maneuvers of free-flying insects. *J. Exp. Biol.* **212**, 1324–1335 (2009).

45. J. W. S. Pringle, The gyroscopic mechanism of the halteres of Diptera. *Philos. Trans. R. Soc. Lond. B Biol. Sci.* **233**, 347–384 (1948).

46. G. Nalbach, R. Hengstenberg, The halteres of the blowfly Calliphora. *J. Comp. Physiol. A* **175**, 695–708 (1994).

47. T. L. Mohren, T. L. Daniel, A. L. Eberle, P. G. Reinhall, J. L. Fox, Coriolis and centrifugal forces drive haltere deformations and influence spike timing. *J. R. Soc. Interface* **16**, 20190035 (2019).

48. B. H. Dickerson, A. M. de Souza, A. Huda, M. H. Dickinson, Flies regulate wing motion via active control of a dual-function gyroscope. *Curr. Biol.* **29**, 3517–3524.e3 (2019).

49. J. J. Wine, F. B. Krasne, The cellular organization of crayfish escape behavior. *Biol. Crustacea* **4**, 241–292 (1982).

50. A. Hess, Vertebrate slow muscle fibers. *Physiol. Rev.* **50**, 40–62 (1970).

51. D. J. Aidley, *The Physiology of Excitable Cells* (Cambridge Univ. Press, ed. 4, 2012).

52. J. Isaacman-Beck, K. C. Paik, C. F. Wienecke, H. H. Yang, Y. E. Fisher, I. E. Wang, I. G. Ishida, G. Maimon, R. I. Wilson, T. R. Clandinin, SPARC enables genetic manipulation of precise proportions of cells. *Nat. Neurosci.*, **23 23**, 1168–1175 (2020).

53. G. Heide, Neural mechanisms of flight control in Diptera. *BIONA Rep.* **2**, 35–52 (1983).

54. J. S. Phelps, D. G. C. Hildebrand, B. J. Graham, A. T. Kuan, L. A. Thomas, T. M. Nguyen, J. Buhmann, A. W. Azevedo, A. Sustar, S. Agrawal, M. Liu, B. L. Shanny, J. Funke, J. C. Tuthill, W. C. A. Lee, Reconstruction of motor control circuits in adult Drosophila using automated transmission electron microscopy. *Cell* **184**, 759–774.e18 (2021).

55. T. R. Shirangi, A. M. Wong, J. W. Truman, D. L. Stern, Doublesex regulates the connectivity of a neural circuit controlling drosophila male courtship song. *Dev. Cell* **37**, 533–544 (2016).

56. M. T. Ke, S. Fujimoto, T. Imai, SeeDB: A simple and morphology-preserving optical clearing agent for neuronal circuit reconstruction. *Nat. Neurosci.* **16**, 1154–1161 (2013).

57. D. H. Theriault, N. W. Fuller, B. E. Jackson, E. Bluhm, D. Evangelista, Z. Wu, M. Betke, T. L. Hedrick, A protocol and calibration method for accurate multi-camera field videography. *J. Exp. Biol.* **217**, 1843–1848 (2014).

58. S. P. Sane, M. H. Dickinson, The aerodynamic effects of wing rotation and a revised quasi-steady model of flapping flight. *J. Exp. Biol.* **205**, 1087–1096 (2002).

59. M. P. Suver, A. Huda, N. Iwasaki, S. Safarik, M. H. Dickinson, An array of descending visual interneurons encoding self-motion in Drosophila. *J. Neurosci.* **36**, 11768–11780 (2016).

60. J. P. Whitney, R. J. Wood, Aeromechanics of passive rotation in flapping flight. *J. Fluid Mech.* **660**, 197–220 (2010).

61. S. N. Fry, R. Sayaman, M. H. Dickinson, The aerodynamics of hovering flight in Drosophila. *J. Exp. Biol.* **208**, 2303–2318 (2005).

62. C. P. Ellington, The aerodynamics of hovering insect flight. II. Morphological parameters. *Philos. Trans. R. Soc. B Biol. Sci.* **305**, 17–40 (1984).

63. Z. J. Wang, J. M. Birch, M. H. Dickinson, Unsteady forces and flows in low Reynolds number hovering flight: Two-dimensional computations vs robotic wing experiments. *J. Exp. Biol.* **207**, 449–460 (2004).

64. J. M. Birch, M. H. Dickinson, Spanwise flow and the attachment of the leading-edge vortex on insect wings. *Nature* **412**, 729–733 (2001).

65. R. M. Noest, Z. Jane Wang, Optimal wing hinge position for fast ascent in a model fly. *J. Fluid Mech.* **849**, 498–509 (2018).

66. E. D. Tytell, P. Holmes, A. H. Cohen, Spikes alone do not behavior make: Why neuroscience needs biomechanics. *Curr. Opin. Neurobiol.* **21**, 816–822 (2011).

67. N. J. Cowan, E. S. Fortune, The critical role of locomotion mechanics in decoding sensory systems. *J. Neurosci.* **27**, 1123–1128 (2007).

68. B. Cheng, S. N. Fry, Q. Huang, W. B. Dickson, M. H. Dickinson, X. Deng, Turning dynamics and passive stability in flapping flight, in *Proceedings of the IEEE International Conference on Robotics and Automation (ICRA)* (IEEE, 2009) pp. 1–8.

69. N. J. Cowan, J. Lee, R. J. Full, Task-level control of rapid wall following in the American cockroach. *J. Exp. Biol.* **209**, 1617–1629 (2006).

70. M. H. Dickinson, Haltere-mediated equilibrium reflexes of the fruit fly, Drosophila melanogaster. *Philos. Trans. R. Soc. Lond. B Biol. Sci.* **354**, 903–916 (1999).

71. M. J. Elzinga, W. B. Dickson, M. H. Dickinson, The influence of sensory delay on the yaw dynamics of a flapping insect. *J. R. Soc. Interface* **9**, 1685–1696 (2012).

72. R. Court, S. Namiki, J. D. Armstrong, J. Börner, G. Card, M. Costa, M. Dickinson, C. Duch, W. Korff, R. Mann, D. Merritt, R. K. Murphey, A. M. Seeds, T. Shirangi, J. H. Simpson, J. W. Truman, J. C. Tuthill, D. W. Williams, D. Shepherd, A systematic nomenclature for the drosophila ventral nerve cord. *Neuron* **107**, 1071–1079.e2 (2020).

73. M. H. Dickinson, F. T. Muijres, The aerodynamics and control of free flight manoeuvres in Drosophila. *Philos. Trans. R. Soc. B Biol. Sci.* **371**, 20150388 (2016).
